# ROS‐Responsive Nanoparticle Delivery of Dexmedetomidine Protects the Gut Vascular Barrier After Intestinal Ischemia/Reperfusion via the HDAC‐H3K27ac‐TCF4 Axis

**DOI:** 10.1002/advs.77702

**Published:** 2026-09-08

**Authors:** Hu‐Fei Zhang, Jian‐Tong Shen, Hua‐Hua Zhang, Si‐Qi Gao, Ji‐Ao Wang, Yue‐Ling Wang, Mei‐Ling Li, Zi‐Meng Liu, Yi‐Nan Zhang, Yan Li, Xu‐Yu Zhang

**Affiliations:** ^1^ Department of Anesthesiology The First Affiliated Hospital Sun Yat‐sen University Guangzhou People's Republic of China; ^2^ College of Chemistry and Materials Science Jinan University Guangzhou People's Republic of China; ^3^ Department of Cardiology The First Affiliated Hospital of Jinan University Guangzhou People's Republic of China; ^4^ Department of Critical Care Medicine The First Affiliated Hospital Sun Yat‐sen University Guangzhou People's Republic of China; ^5^ State Key Laboratory of Frigid Zone Cardiovascular Diseases Harbin Medical University Harbin People's Republic of China

**Keywords:** dexmedetomidine, gut vascular barrier, HDAC, intestinal ischemia/reperfusion, ROS‑responsive nanoparticles, TCF4

## Abstract

Intestinal ischemia/reperfusion (I/R) disrupts the gut vascular barrier (GVB), causing bacterial translocation and organ injury. Dexmedetomidine can protect the GVB, but its clinical use is limited by dose‑dependent cardiorespiratory depression. To address this, we develop reactive oxygen species (ROS)‑responsive nanoparticles for targeted intestinal delivery and evaluate the efficacy and mechanism of dexmedetomidine‑loaded nanoparticles (Dex‑NPs) against intestinal I/R‑induced GVB damage. We observe that GVB disruption in clinical and experimental specimens is associated with reduced expression of Claudin5 and VE‑cadherin. Dex‑NPs preferentially accumulate in the ischemic intestine and release the drug upon ROS stimulation. Low‑dose Dex‑NPs (dexmedetomidine 5 µg/kg) confer GVB and hepatic protection comparable to high‑dose free dexmedetomidine, without detectable acute cardiorespiratory depression under the conditions tested. Mechanistically, dexmedetomidine inhibits histone deacetylase (HDAC), enhances H3K27 acetylation, and upregulates T‐cell factor 4 (TCF4, encoded by *TCF7L2*), which directly binds and activates *CLDN5* and *CDH5* promoters, thereby restoring Claudin5/VE‑cadherin expression and GVB integrity; TCF4 knockdown largely abolishes these protective effects. In summary, ROS‑responsive Dex‑NPs enable low‑dose dexmedetomidine to preserve GVB integrity and mitigate post‑I/R liver injury via HDAC inhibition and TCF4 upregulation, circumventing dose‑limiting toxicity and suggesting TCF4 as a potential therapeutic target in oxidative stress‑associated gut endothelial barrier dysfunction.

## Graphical abstract

A low dose of dexmedetomidine, delivered by ROS‐responsive nanoparticles, preferentially accumulates in the ischemic intestine and restores gut vascular barrier integrity after ischemia/reperfusion injury. This nanoparticle‐based strategy prevents bacterial translocation and liver damage without cardiorespiratory side effects, working through HDAC inhibition and TCF4‐mediated upregulation of junction proteins. ToC image created by BioRender.

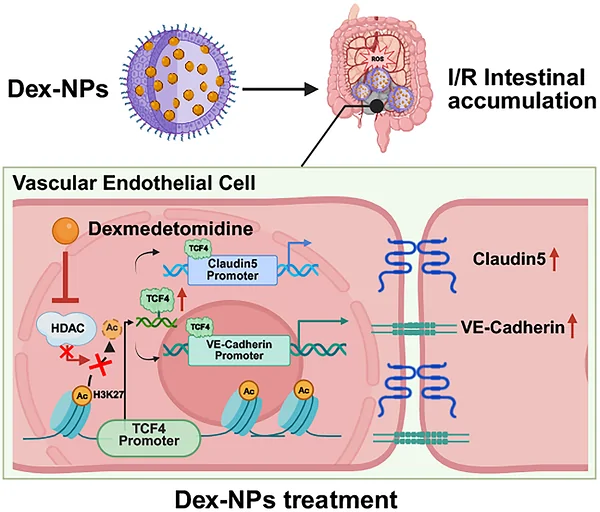

## 1 Introduction

Intestinal ischemia/reperfusion (I/R) injury is a life‐threatening condition frequently encountered in critical care settings, occurring during major vascular procedures (e.g., open abdominal aortic aneurysm repair, mesenteric artery embolectomy), cardiopulmonary bypass, or in conditions that sharply reduce intestinal perfusion (e.g., hemorrhagic shock, severe sepsis, or trauma) [1]. The intestine harbors the body's largest reservoir of bacteria and endotoxins [2], and its protective function depends on an intact barrier [3]. Intestinal I/R markedly disrupts this barrier, permitting translocation of luminal bacteria and toxins to distant organs, which can trigger systemic inflammatory response syndrome (SIRS) and multiple organ dysfunction syndrome (MODS), with reported mortality rates of 67%–80% [4, 5, 6]. Therefore, elucidating the mechanisms underlying I/R‐induced intestinal barrier disruption and identifying effective therapeutic strategies are essential for interrupting this lethal cycle.

The conventional view of the intestinal barrier encompasses mechanical, chemical, immune, and biological components [7]. Spadoni et al. defined the “gut vascular barrier” (GVB) as a novel final line of defense between the intestine and the systemic circulation [8]. Analogous to the blood‐brain barrier, the GVB is formed by intestinal vascular endothelial cells and their intercellular junctions [9]. The principal function of the GVB is to restrict the paracellular passage of macromolecules and bacteria via adherens junctions mediated by VE‐cadherin [10] and tight junctions containing Claudin5 [11, 12]. Despite its recognized importance, the molecular mechanisms by which intestinal I/R disrupts the GVB, particularly whether this injury directly involves downregulation of these junctional proteins, remain incompletely understood.

We have previously demonstrated that dexmedetomidine protects the GVB following intestinal I/R. Notably, this protective effect was dose‐dependent, with only a high‐dose regimen conferring significant barrier protection [13, 14]. However, high‐dose dexmedetomidine induces severe cardiorespiratory depression, including marked decreases in heart rate and respiratory rate, which has been documented in both clinical settings [15, 16] and animal studies [14, 17]. These dose‐limiting toxicities severely restrict the translational potential of dexmedetomidine for this indication. In fact, therapeutically effective doses in animal studies often substantially exceed clinically relevant doses, and such high doses inevitably cause dose‐limiting side effects in clinical practice, thereby contributing to the failure of 92% of drug translation studies [18]. Thus, a central challenge is to achieve targeted delivery of low‐dose dexmedetomidine to the injured GVB, thereby maximizing therapeutic efficacy while minimizing systemic side effects.

To address this challenge, we developed a targeted therapeutic strategy based on reactive oxygen species (ROS)‐responsive nanotechnology. Oxidative stress and the accompanying ROS burst are hallmark pathological features of I/R injury [19, 20], and ROS‐responsive nanomedicines have shown preliminary therapeutic potential in cardiac and cerebral I/R models [21, 22, 23]. Exploiting the ROS‐rich microenvironment of ischemic intestine, we synthesized ROS‐responsive nanoparticles for efficient loading and targeted delivery of dexmedetomidine (Dex‐NPs). We hypothesized that this strategy would concentrate drug release at the ischemic intestine, thereby maximizing local barrier protection while minimizing systemic side effects. In this study, we evaluated the efficacy of Dex‐NPs in protecting the GVB following intestinal I/R and explored the potential mechanisms driving Claudin5 and VE‐cadherin restoration.

## 2 Results

### 2.1 GVB Damage With Downregulation of Claudin5 and VE‑Cadherin After Intestinal I/R

**FIGURE 1:**
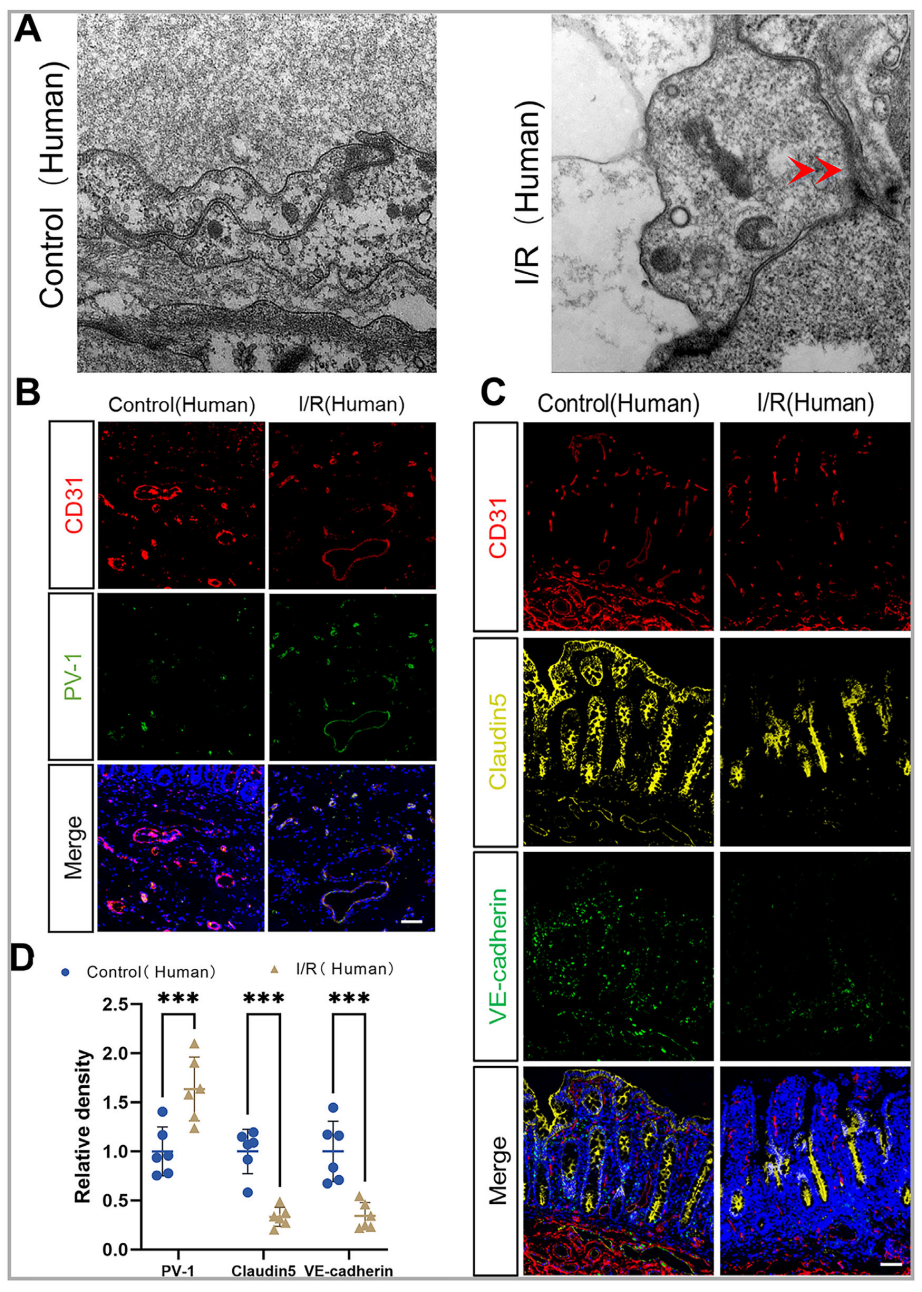
Intestinal I/R injury leads to GVB damage, accompanied by disruption of vascular endothelial cell junctions in human patients. (A) Electron microscopy images showing ultrastructural injury of the GVB in human intestinal I/R patients. Red arrows indicate disrupted endothelial cell junctions in the I/R group. Scale bar: 500 nm. (B) Immunofluorescence analysis of endothelial permeability marker PV‐1 (green) in human ileum sections from control and intestinal I/R patients (200×). CD31 (red) labels endothelial cells, and nuclei are counterstained with DAPI (blue). Scale bar: 100 µm. (C) Immunofluorescence staining of Claudin5 (yellow, tight junctions) and VE‐cadherin (green, adherens junctions) in human ileum sections (200×), CD31 (red), DAPI (blue). Scale bar: 100 µm.(D) Quantitative analysis of the colocalized fluorescence intensity of PV‑1, Claudin5, and VE‑cadherin with CD31 in control and intestinal I/R human intestinal tissues. Data are presented as mean ± SD (*n* = 6). ^***^ *p* < 0.001. I/R, intestinal ischemia/reperfusion; GVB, gut vascular barrier; PV‐1, plasmalemma vesicle‐associated protein‐1; DAPI, 4,6‐diamino‐2‐phenyl indole; SD, standard deviation.

To evaluate the structural damage of the GVB following intestinal I/R injury, we first examined intestinal tissues from I/R patients. Electron microscopy revealed marked ultrastructural disruption of endothelial cell junctions in the GVB of I/R patients compared with controls (Figure 1A). Immunofluorescence analysis further showed that plasmalemma vesicle‐associated protein‐1(PV‐1), a permeability marker, was significantly increased in ileum sections from I/R patients, while the tight junction protein Claudin5 and the adherens junction protein VE‐cadherin were substantially reduced (Figure 1B–D).

**FIGURE 2:**
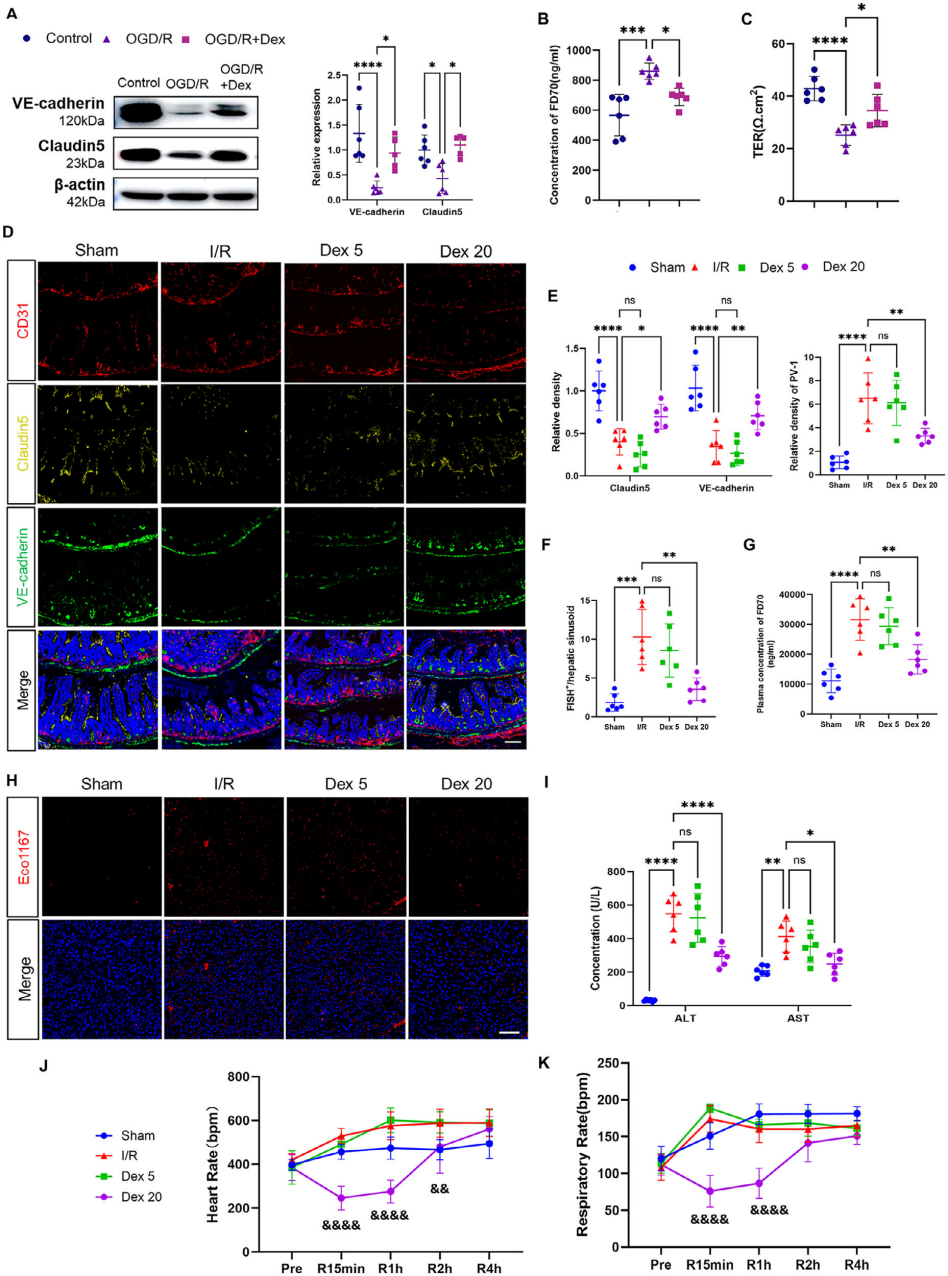
High‐dose dexmedetomidine ameliorates GVB injury after intestinal I/R but induces side effects, whereas low dose is ineffective. (A–C) In vitro effects of dexmedetomidine on OGD/R‐induced endothelial barrier impairment in HUVECs. (A) Western blot analysis of VE‐cadherin and Claudin5 protein levels. (B) Endothelial monolayer permeability to FD70. (C) TER of HUVECs monolayers. (D‐J) Mice were treated with PBS (Sham/I/R) or different doses of dexmedetomidine (Dex 5, 5 µg/kg; Dex 20, 20 µg/kg) and assessed 4 h after reperfusion. (D) Representative immunofluorescence images of ileum sections showing CD31 (red), Claudin5 (yellow), and VE‐cadherin (green). Nuclei are counterstained with DAPI (blue). Scale bar: 100 µm. (E) Quantitative analysis of the relative density of Claudin5, VE‐cadherin, and PV‐1 with CD31 in ileum sections. (F) Quantification of FISH‐positive bacteria in hepatic sinusoids. (G) Plasma concentration of FD70. (H) Liver sections stained by FISH using an *E. coli* probe (Eco1167, red), Nuclei are counterstained with DAPI (blue). Scale bar: 100 µm. (I) Serum levels of ALT and AST. (J) Dynamic changes in heart rate. (K) Dynamic changes in respiratory rate. Data are presented as mean ± SD (*n* = 6). ^*^ *p* < 0.05, ^**^ *p* < 0.01, ^***^ *p* < 0.001, ^****^ *p* < 0.0001, ns, not significant; ^&&^ *p* < 0.01, ^&&&&^ *p* < 0.0001 vs. I/R group. GVB, gut vascular barrier; I/R, intestinal ischemia/reperfusion; PV‐1, plasmalemma vesicle‐associated protein‐1; DAPI, 4,6‐diamidino‐2‐phenylindole; FISH, fluorescence in situ hybridization; *E. coli*, Escherichia coli; FD70, FITC‐dextran 70; OGD/R, oxygen‐glucose deprivation/reperfusion; HUVECs, human umbilical vein endothelial cells; TER, transendothelial electrical resistance; ALT, alanine aminotransferase; AST, aspartate aminotransferase.

Consistent changes were also detected in an oxygen‑glucose deprivation/reoxygenation (OGD/R) model using human umbilical vein endothelial cells (HUVECs). The downregulation of Claudin5 and VE‐cadherin led to increased endothelial barrier permeability and a decrease in transendothelial electrical resistance (TER) (Figure 2A–C). Furthermore, similar disruptions in the GVB were observed in mice undergoing intestinal I/R. The current experiments demonstrated that at 4 h after reperfusion, downregulation of Claudin5 and VE‑cadherin, increased PV‑1 expression, enhanced translocation of intestinal bacteria and macromolecules, and elevated serum alanine aminotransferase (ALT) and aspartate aminotransferase (AST) levels were observed (Figure 2D–I and Figure S1A). Additionally, Chiu's histological scoring confirmed significant mucosal injury in the I/R group compared with sham controls (Figure S1C).

Collectively, these findings indicate that intestinal I/R injury disrupts endothelial cell junctions by inducing downregulation of Claudin5 and VE‐cadherin, and therefore leads to GVB damage and dysfunction.

### 2.2 High‐Dose Dexmedetomidine Ameliorates GVB Damage but Causes Cardiorespiratory Depression

Dexmedetomidine is a commonly used sedative in critically ill patients, and our previous studies have demonstrated its protective effects on GVB after intestinal I/R injury [13]. In the present study, we further investigated its impact on Claudin5 and VE‐cadherin expression both in vitro and in vivo.

In vitro, the current results demonstrated that dexmedetomidine protected the endothelial barrier. Specifically, it restored protein levels of Claudin5 and VE‐cadherin, reduced monolayer permeability to FITC‐Dextran 70 (FD70), and increased TER, indicating enhanced endothelial integrity (Figure 2A–C).

In vivo, based on our previous results [13], a low or high dose (Dex5 or Dex20) of dexmedetomidine was administered. The data showed that a high dose of dexmedetomidine (20 µg/kg) effectively restored the barrier junction proteins Claudin5 and VE‐cadherin, and significantly reduced Chiu's histological score (Figure S1C), reduced bacterial translocation to the liver, and lowered plasma FD70, ALT, and AST levels at 4 h after reperfusion, indicating enhanced GVB integrity and reduced liver injury (Figure 2D‐I and Figure S1A). Despite these protective effects, 20 µg/kg of dexmedetomidine induced significant cardiovascular and respiratory side effects, including decreased heart rate and respiration rate over time. In contrast, a low dose (5 µg/kg) of dexmedetomidine, a dose equivalent to that commonly used in clinical practice, did not significantly depress cardiopulmonary function (Figure 2J–K), whereas it delivered minor protective effects for GVB and liver in mice (Figure 2D–I). Furthermore, two deaths occurred in the Dex20 group soon after injection (Figure S1B), underscoring the serious risk associated with the cardiopulmonary depression induced by the high dose.

These findings highlight that the protective effect of dexmedetomidine on the GVB is dose‐dependent; whereas high‐dose dexmedetomidine is often accompanied by cardiorespiratory side effects, which severely limit its clinical use.

### 2.3 Synthesis, Characterization, and Biocompatibility Evaluation of Polymeric Nanomedicines

Given that high‐dose dexmedetomidine provides organ protection but causes cardiorespiratory complications, likely due to its distribution to non‑target organs, we sought to develop a strategy for targeted delivery specifically to the ischemic intestine. Ischemic tissues are known to produce high levels of ROS. In this study, compared with sham‑operated mice, intestinal I/R‑injured mice exhibited a marked increase in ROS production exclusively in the intestine, whereas the kidney, lung, spleen, liver, and heart showed no significant ROS elevation (Figure S2), indicating a local, intestinal‑specific oxidative stress response. Leveraging this ROS‑rich microenvironment in the intestinal tissue, we therefore developed ROS‑responsive nanoparticles to encapsulate dexmedetomidine.

**FIGURE 3:**
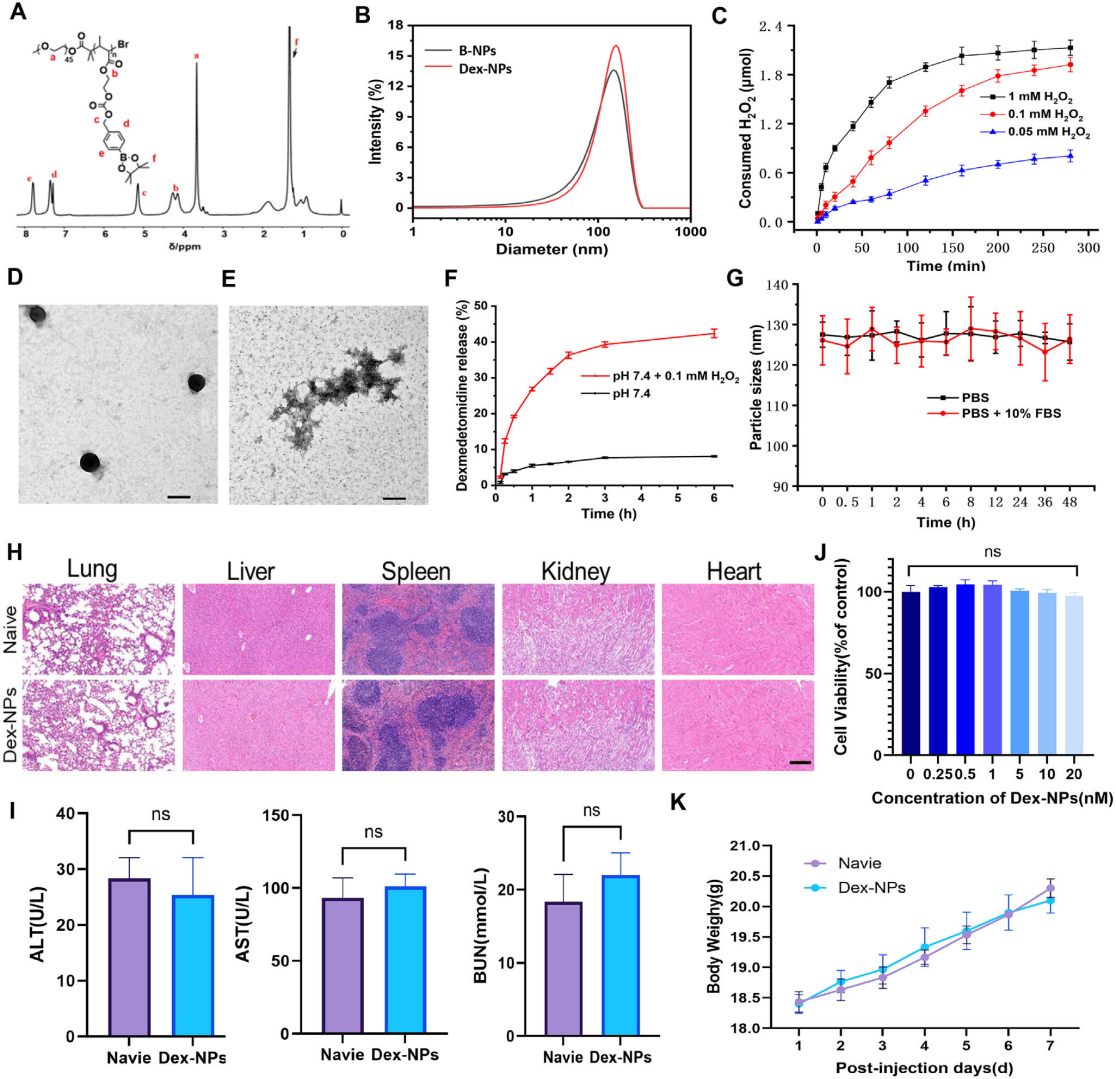
Characterization and biocompatibility assessment of polymeric nanomedicines. (A) ^1^H NMR spectrum of mPEG‐PPBEM in CDCl_3_. (B) The particle size distribution of B‐NPs and Dex‐NPs at pH 7.4 determined by DLS. (C) Consumed H_2_O_2_ after incubation of B‐NPs (PBEM unit, 0.1 × 10^−^^3^ M) in various concentrations of H_2_O_2_ solution (0.05, 0.1, and 1 mM) for different time points at 37°C. The data were monitored by a UV–vis spectrometer at 375 nm. (D,E) TEM images of nanomedicines at pH 7.4 (D) and pH 7.4 + 0.1 mM H_2_O_2_ (E), Scale bars: 200 nm. (F) In vitro drug release from Dex‐NPs at pH 7.4 and pH 7.4 + 0.1 mM H_2_O_2_. (G) Stability of nanoparticles in PBS and PBS + 10% FBS measured by DLS. (H) Histopathological analysis of lung, liver, spleen, kidney, and heart sections stained with H&E. Top row: Naive. Bottom row: Dex‐NPs. Scale bar: 100 µm. (I) Serum levels of biochemical markers for hepatic function (AST, ALT) and renal function (BUN). (J) Effect of Dex‐NPs concentration on HUVECs viability following OGD/R. (K) Body weight changes in mice over 7 days following injection of Dex‐NPs. Data are presented as mean ± SD (*n* = 3). ns, not significant. B‐NPs, blank nanoparticles; Dex‐NPs, dexmedetomidine‑loaded nanoparticles; DLS, dynamic light scattering; TEM, transmission electron microscopy; H&E, hematoxylin and eosin; AST, aspartate aminotransferase; ALT, alanine aminotransferase; BUN, blood urea nitrogen; HUVECs, human umbilical vein endothelial cells; OGD/R, oxygen‐glucose deprivation/reperfusion.

The nanomedicines were assembled from mPEG‐PPBEM with dexmedetomidine loaded in the hydrophobic micellar core, and the resulting formulation was denoted as Dex‐NPs. Firstly, the ROS‐sensitive polymer mPEG‐PPBEM was synthesized via multiple steps as shown in Figure S3A, and the successful synthesis of the designed polymer was verified by ^1^H‐NMR spectroscopy and FT‐IR analysis (Figure S3B–F). Specifically, after activation with 1,1′‐carbonyldiimidazole (CDI), the 4‐(hydroxymethyl) phenylboronic acid pentaerythritol ester (HMPBE) derivative showed disappearance of the hydroxyl characteristic signal and appearance of new signals at 7.03, 7.65, and 8.37 ppm corresponding to the CDI groups. After the reaction with methyl methacrylate hydroxyethyl ester, the characteristic peaks of the CDI hydrogens disappeared, and the characteristic signals of methyl methacrylate hydroxyethyl ester appeared at 1.87, 4.31, and 6.01 ppm, verifying the successful synthesis of the PBEM monomer. After mPEG‐Br was synthesized, mPEG‐PPBEM was generated through atom transfer radical polymerization (ATRP), and the characteristic signal of carbon–carbon double bonds in PBEM disappeared, while the characteristic signals of boronic acid pentaerythritol ester appeared. In addition, all the chemical shifts matched well with the chemical structure of the polymer, indicating the successful synthesis of mPEG‐PPBEM. Moreover, based on the integral values of the characteristic peaks of the phenyl group of PPBEM and the methylene groups of mPEG, the degree of polymerization of PPBEM was 25 (Figure 3A).

Then, mPEG‐PPBEM was self‐assembled into micelles, incorporating dexmedetomidine via hydrophobic interactions. The critical micelle concentration (CMC) of mPEG‑PPBEM in aqueous solution was determined to be 4.3 µg/mL using the pyrene fluorescence probe method (Figure S3H). The particle sizes and zeta potentials of the nanoparticles were measured by dynamic light scattering (DLS). As shown in Figure 3B, the blank nanoparticles (B‐NPs) and drug‐loaded nanoparticles (Dex‐NPs) exhibited diameters of 120 ± 3 nm and 130 ± 6 nm, respectively. The drug loading content and encapsulation efficiency of Dex‑NPs were determined to be 7.10 ± 0.31% and 76.38 ± 4.6%, respectively. The polydispersity index (PDI) of B‑NPs and Dex‑NPs was 0.274 and 0.263, respectively. To assess batch‑to‑batch reproducibility, three independent batches were prepared using the same formulation, and the drug loading content varied within 0.5%. In addition, the zeta potentials of the two nanoparticles were −2.4 ± 1.7 mV and −2.2 ± 0.42 mV (Figure S3G). The weakly negative zeta potentials may be attributed to the hydrolysis of boronic ester groups [24]. The particle size of approximately 120–130 nm and near‐neutral zeta potential were appropriate for long‐term circulation.

The influence of hydrogen peroxide concentration on the hydrolysis of the PBEM moiety in the polymer was investigated. As depicted in Figure 3C, H_2_O_2_ was rapidly consumed within 100 min, with the rate depending on the initial H_2_O_2_ concentration. A higher initial H_2_O_2_ level resulted in more rapid consumption, indicating accelerated PBEM hydrolysis. By 240 min, the oxidation process was complete at 1 mM H_2_O_2_, while reaching 93% and 89% completion at 0.1 mM and 0.05 mM H_2_O_2_, respectively. These findings confirmed that the hydrogen peroxide consumption capacity of the polymer is dependent on the available H_2_O_2_ concentration.

Additionally, TEM observation showed that Dex‐NPs presented a spherical morphology with a diameter close to 130 nm (Figure 3D), which was consistent with the DLS results. The formation of nanoparticles is mainly driven by hydrophobic interactions between the polymer and drug molecules. Thus, the removal of the hydrophobic phenylboronic acid pinacol ester triggered by ROS can induce nanoparticle disassembly [25], which was verified by TEM observation of Dex‐NPs degradation under H_2_O_2_‐containing conditions (Figure 3E). The disassembly of Dex‐NPs may trigger efficient drug release; therefore, in vitro release experiments were conducted at pH 7.4 and pH 7.4 plus 100 µM H_2_O_2_. As expected, dexmedetomidine was hardly released at pH 7.4, while it was rapidly released from nanoparticles in ROS‐rich conditions. Dex‐NPs exhibited a rapid release profile in the presence of ROS (pH 7.4 + 0.1 mM H_2_O_2_), with a cumulative release of approximately 42% within 6 h. In contrast, the release of dexmedetomidine was less than 10% at pH 7.4(Figure 3F). In addition, the lysosomal acidic environment might accelerate drug release [26]. Most importantly, particle size tests in fetal bovine serum (FBS)‐containing conditions indicated that Dex‐NPs could maintain their original size in the physiological environment (Figure 3G). These results demonstrated that our nanomedicine is stable during circulation and can release the encapsulated drug in ROS‐rich pathological sites.

To evaluate the clearance profile of the polymer carrier, mPEG‑PPBEM was labeled with salicyl fluoresone and self‑assembled into SF‑NPs, which were then injected into C57BL/6J mice. As shown in Figure S3I, the fluorescence intensity in the blood decreased rapidly over time, with approximately 60% of the polymer cleared from circulation within 6 h and reaching baseline levels by 24 h postinjection, indicating that the mPEG‑PPBEM carrier is efficiently cleared from the bloodstream. To further assess the biocompatibility of Dex‑NPs, healthy mice received tail vein injections of Dex‐NPs, while control mice received PBS. H&E staining of lung, liver, spleen, kidney, and heart sections following Dex‐NPs treatment revealed no obvious abnormalities (Figure 3H). Consistently, serum biochemical assays measuring hepatic markers (AST, ALT) and the renal marker (blood urea nitrogen, BUN) showed no notable changes one week after daily nanoparticle administration (Figure 3I). Meanwhile, body weight changes in mice were recorded over a 7‐day period, and no significant differences were observed between groups (Figure 3K). Importantly, Dex‐NPs did not affect HUVECs viability after OGD/R at all tested concentrations (Figure 3J). Collectively, these results demonstrate that Dex‐NPs possess favorable biocompatibility both in vivo and in vitro.

### 2.4 Dex‐NPs Selectively Accumulate in the Ischemic Intestine and Protect GVB Integrity After Intestinal I/R Injury

**FIGURE 4:**
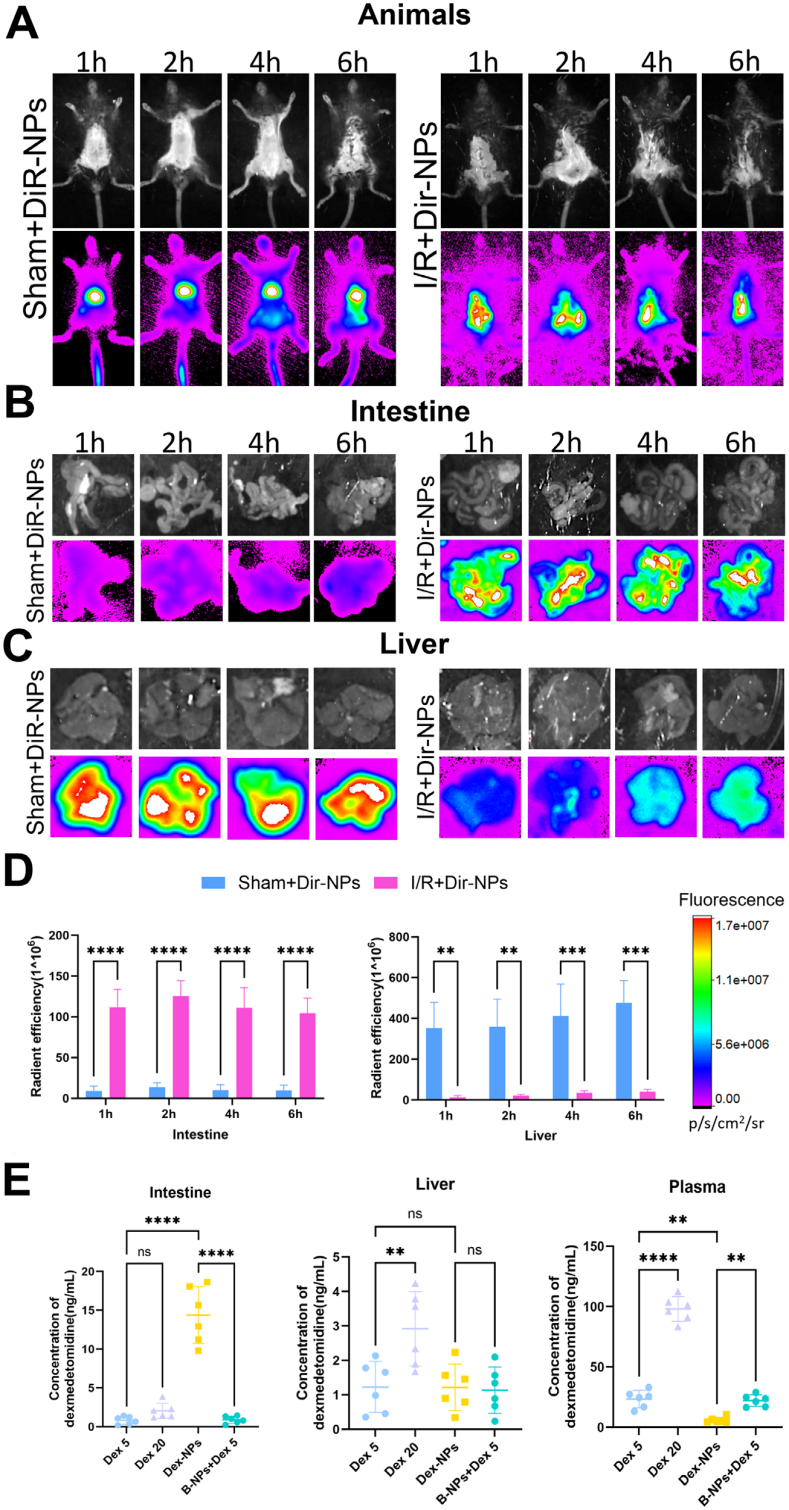
In vivo and ex vivo targeting of Dex‑NPs to ischemic tissues, and pharmacokinetic quantification of dexmedetomidine distribution. (A–D) Mice were intravenously injected with DiR‑labeled nanoparticles (DiR‑NPs). (A) In vivo whole‑body fluorescence imaging of sham‑operated and intestinal I/R mice at 1, 2, 4, and 6 h postinjection. (B) Ex vivo imaging of the intestines harvested from the same mice at the indicated time points. (C) Ex vivo imaging of the livers. (D) Quantitative analysis of fluorescence signals from the intestines and livers shown in (B) and (C). Data are presented as mean ± SD (*n* = 3). The pseudocolor scale indicates fluorescence radiant efficiency (red, high; purple, low). (E) LC‑MS/MS quantification of dexmedetomidine concentrations in plasma, intestinal, and liver tissues at 1 h post‑administration. Data are presented as mean ± SD (*n* = 6). ^**^ *p* < 0.01, ^***^ *p* < 0.001, ^****^ *p* < 0.0001, ns, not significant.

Given the local, intestinal‑specific oxidative stress response following I/R injury (Figure S2), we next evaluated the ability of our nanoparticles to target ischemic tissue in a mouse I/R model using DiR‑labeled nanoparticles (DiR‑NPs). DiR‑NPs were intravenously injected into sham‑operated and intestinal I/R mice. In vivo and ex vivo fluorescence imaging revealed selective accumulation of DiR‑NPs in the ischemic intestine of I/R mice, but not in other organs (Figure 4A–D and Figure S4A). In contrast, in sham‑operated mice, DiR‑NPs mainly accumulated in the liver, likely due to hepatic metabolism, and only minimal signals were detected in the intestines (Figure 4A–D). Moreover, in renal I/R mice, intense fluorescence signals were observed in the ischemic kidney (Figure S4B). These findings confirmed the effective preferential accumulation of the synthesized nanoparticles to ischemic tissues.

**FIGURE 5:**
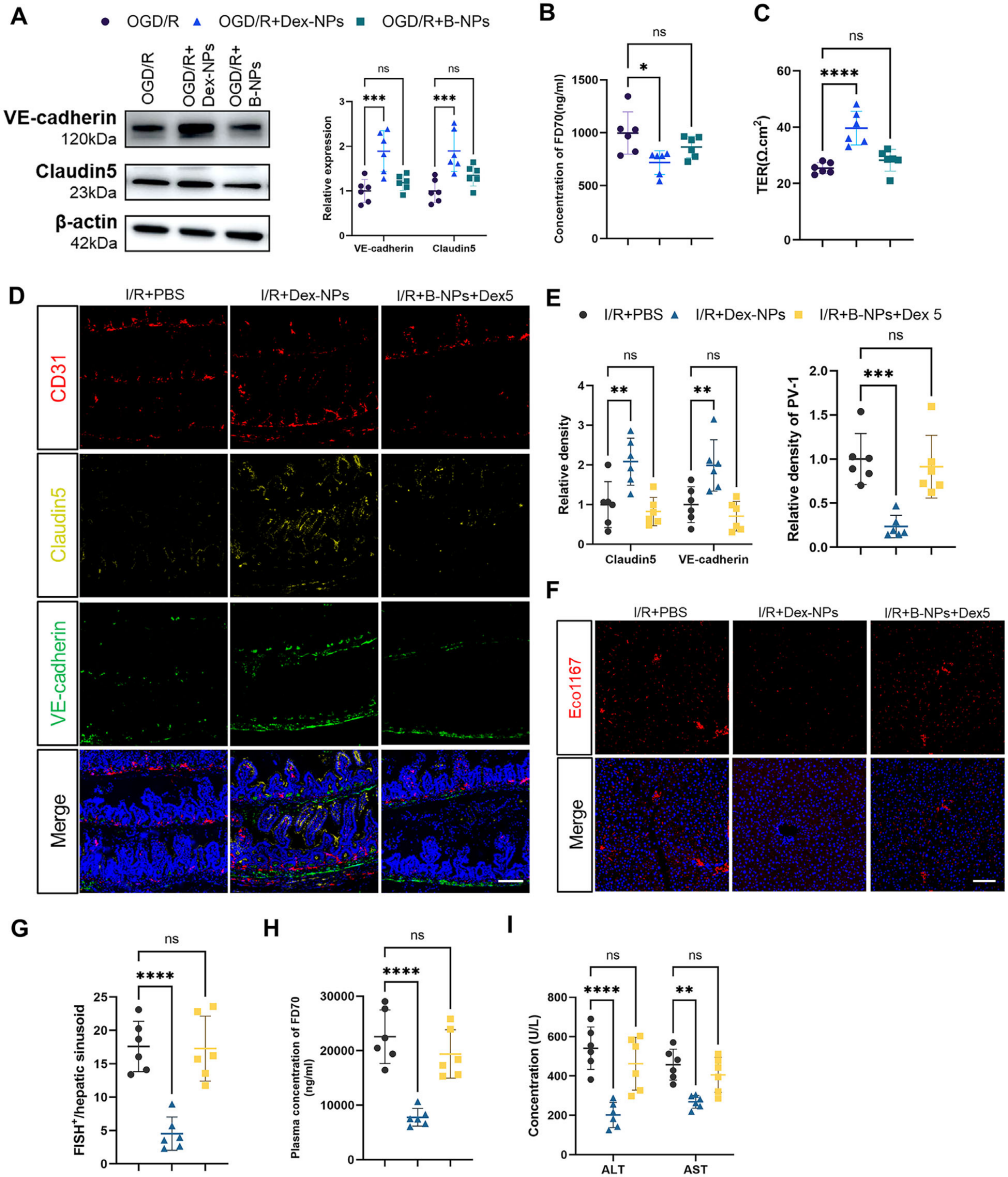
Dex‐NPs protect GVB integrity after intestinal I/R injury. (A–C) In vitro effects of Dex‐NPs and B‐NPs on OGD/R‐induced endothelial barrier impairment in HUVECs. (A) Western blot analysis of VE‐cadherin and Claudin5 protein levels. (B) Endothelial monolayer permeability to FD70. (C) TER of HUVECs monolayers. (D–I) Mice subjected to intestinal I/R were treated with PBS (I/R+PBS), Dex‐NPs, or B‐NPs + Dex 5. (D) Representative immunofluorescence images of ileum sections showing CD31 (red), Claudin5 (yellow), and VE‐cadherin (green). Nuclei are counterstained with DAPI (blue). Scale bar: 100 µm. (E) Quantitative analysis of the relative fluorescence density of Claudin5, VE‐cadherin, and PV‐1 with CD31 in ileum sections. (F) Liver sections stained by FISH using an *E. coli* probe (Eco1167, red). Nuclei are counterstained with DAPI (blue). Scale bar: 100 µm. (G) Quantification of FISH‐positive bacteria in hepatic sinusoids. (H) Plasma concentration of FD70. (I) Serum levels of ALT and AST. Data are presented as mean ± SD (*n* = 6). ^*^ *p* < 0.05, ^**^ *p* < 0.01, ^***^ *p* < 0.001, ^****^ *p* < 0.0001; ns, not significant. B‐NPs, blank nanoparticles; Dex‐NPs, dexmedetomidine‑loaded nanoparticles; GVB, gut vascular barrier; I/R, intestinal ischemia/reperfusion; PV‐1, plasmalemma vesicle‐associated protein‐1; DAPI, 4,6‐diamidino‐2‐phenylindole; FISH, fluorescence in situ hybridization; *E. coli*, Escherichia coli; FD70, FITC‐dextran 70; OGD/R, oxygen‐glucose deprivation/reperfusion; HUVECs, human umbilical vein endothelial cells; TER, transendothelial electrical resistance; ALT, alanine aminotransferase; AST, aspartate aminotransferase.

To directly quantify the tissue distribution of dexmedetomidine, we performed LC‑MS/MS analysis of plasma, intestinal, and liver samples at 1 h post‑administration. As shown in Figure 4E, the dexmedetomidine concentration in the intestinal tissue was significantly higher in the Dex‑NPs (dexmedetomidine 5 µg/kg) group than in all other groups. In contrast, systemic exposure in the Dex‑NPs group was significantly lower than that in the 20 µg/kg free drug group, and even lower than that in the 5 µg/kg free drug group. These data confirm that nanoparticle encapsulation enables efficient drug enrichment in the ischemic intestine while minimizing systemic distribution, providing a pharmacokinetic basis for the improved safety profile of Dex‑NPs. Subsequently, we investigated whether Dex‑NPs (dexmedetomidine 5 µg/kg) could protect the GVB and mitigate organ damage following intestinal I/R injury. In vitro, in HUVECs subjected to OGD/R, Dex‑NPs treatment increased protein levels of Claudin5 and VE‑cadherin, decreased FD70 permeability, and elevated TER compared with the control group (Figure 5A–C). No protective effects were observed in cells treated with blank nanoparticles alone. In vivo, mice subjected to intestinal I/R were treated with vehicle (I/R + PBS), Dex‑NPs, or blank nanoparticles combined with low‑dose dexmedetomidine (B‑NPs + Dex5; blank nanoparticles and free dexmedetomidine were injected separately). Notably, the combination treatment (B‑NPs + Dex5) failed to confer protection to the GVB. In contrast, treatment with Dex‑NPs significantly restored Claudin5 and VE‑cadherin expression, reduced PV‑1 levels in the intestine (Figure 5D,E, Figure S5A), and concomitantly decreased hepatic bacterial translocation as well as plasma FD70, ALT, and AST levels (Figure 5F–I). Consistently, Chiu's histological scoring confirmed that Dex‑NPs significantly reduced intestinal mucosal injury compared with the I/R group, whereas B‑NPs + Dex5 showed only a modest effect (Figure S5E). Moreover, neither Dex‑NPs nor B‑NPs + Dex5 significantly affected cardiopulmonary function compared with I/R + PBS (Figure S5C,D), nor did they cause any mortality after injection (Figure S5B).

Collectively, these findings demonstrate that the therapeutic benefit of Dex‑NPs relies on nanoparticle‑mediated accumulation and localized release of dexmedetomidine in the ischemic intestine, where dexmedetomidine exerts its barrier‑protective action. Blank nanoparticles are ineffective. Thus, Dex‑NPs effectively preserve GVB integrity following intestinal I/R injury.

### 2.5 Dexmedetomidine Protects GVB by Upregulating Claudin5 and VE‑Cadherin via T‐Cell Factor 4

**FIGURE 6:**
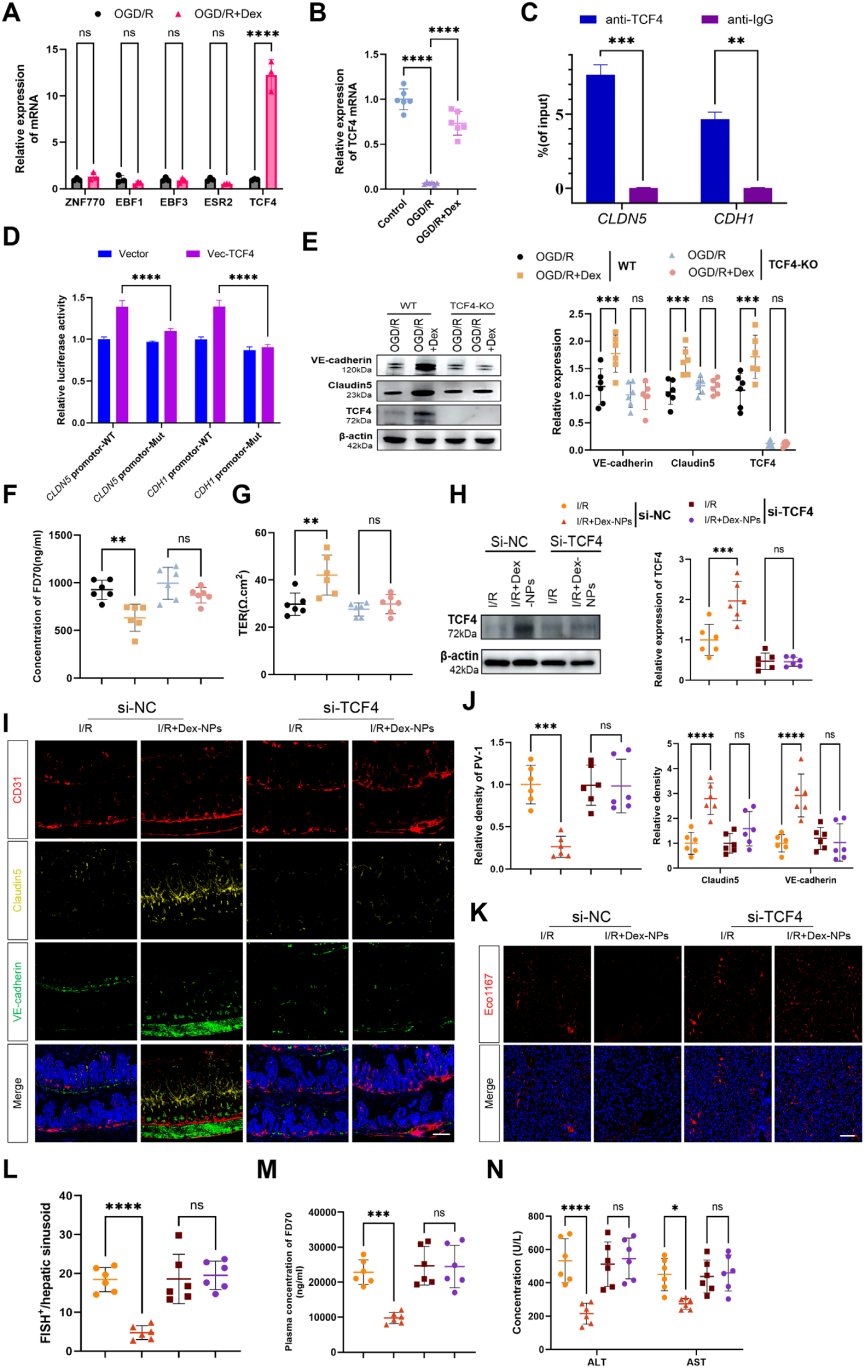
Dexmedetomidine ameliorates GVB injury after intestinal I/R by upregulating Claudin5 and VE‑cadherin via TCF4. (A) qPCR analysis of the mRNA expression of five predicted co‐transcription factors (ZNF770, EBF1, EBF3, ESR2, and TCF4) for Claudin5 and VE‑cadherin. Dexmedetomidine treatment significantly upregulated TCF4 expression compared to the OGD/R group. (B) qPCR analysis of TCF4 mRNA expression in the indicated groups (Control, OGD/R, OGD/R + Dex). (C) ChIP assay showing TCF4 binding to the *CLDN5*(Claudin5) and *CDH5*(VE‐cadherin) promoter regions. (D) Dual‑luciferase reporter assay assessing the effect of TCF4 on *CLDN5* and *CDH5* promoter activity. (E–G) In vitro validation using TCF4‑KO cells: The protective effects of dexmedetomidine on HUVECs barrier function were abolished in TCF4‑KO cells under OGD/R. (E) Western blot analysis of VE‑cadherin, Claudin5, and TCF4 protein levels in WT and TCF4‑KO cells under OGD/R conditions with or without dexmedetomidine treatment. (F,G) FD70 concentration and TER in WT and TCF4‑KO cells under OGD/R ± Dex. (H–N) In vivo validation using TCF4‑knockdown mice: TCF4 knockdown in vivo attenuated the therapeutic efficacy of Dex‑NPs against I/R‑induced GVB injury. (H) Western blot analysis of TCF4 knockdown efficiency in si‑NC vs. si‑TCF4 mice after I/R. (I) Immunofluorescence analysis of Claudin5 (yellow), VE‐cadherin (green) in ileum sections (200×), CD31 (red) DAPI (blue). Scale bars: 100 µm. (J) Quantitative analysis of the relative fluorescence density of Claudin5, VE‐cadherin and PV‐1 with CD31 in ileum sections. (K) Liver sections stained by FISH using an *E. coli* probe (red) (200×), DAPI (blue). Scale bar: 100 µm. (L) Quantification of bacterial presence in the hepatic sinusoid. (M) Plasma levels of FD70 in different groups. (N) Plasma levels of ALT and AST in different groups. Data are presented as mean ± SD (*n* = 6). Statistical significance: ^*^ *p* < 0.05, ^**^ *p* < 0.01, ^***^ *p* < 0.001, ^****^ *p* < 0.0001; ns, not significant. qPCR, quantitative real‐time PCR; ChIP, chromatin immunoprecipitation; KO, knockout; HUVECs, human umbilical vein endothelial cells; OGD/R, oxygen‐glucose deprivation/reperfusion; WT, wild‑type; FD70, FITC‐dextran 70; TER, transendothelial electrical resistance; Dex‐NPs, dexmedetomidine‑loaded nanoparticles; PV‐1, plasmalemma vesicle‐associated protein‐1; DAPI, 4,6‐diamidino‐2‐phenylindole; FISH, fluorescence in situ hybridization; *E. coli*, Escherichia coli.

The nanocarrier precisely delivers dexmedetomidine to the ischemic intestine. However, the mechanism by which dexmedetomidine protects the intestinal barrier under pathological conditions remains unknown. To elucidate the mechanism through which dexmedetomidine upregulates the key junctional proteins Claudin5 and VE‑cadherin, we performed an integrated series of bioinformatic and experimental investigations. Potential common transcriptional regulators of *CLDN5* (encoding Claudin5) and *CDH5* (encoding VE‑cadherin) were predicted using multiple databases and tools, including GTRD, TFDB, and two JASPAR‑based analyses (PWMEnrich and FIMO). The overlapping candidates included ZNF770, EBF1, EBF3, ESR2, and T‐cell factor 4 (TCF4, encoded by *TCF7L2*) (Figure S6A). Subsequent cell experiments revealed that TCF4 mRNA expression was significantly increased in cells treated with dexmedetomidine following OGD/R (Figure 6A), therefore, we selected TCF4 as the key transcription factor for subsequent studies.

In vitro, TCF4 mRNA expression was significantly decreased in HUVECs following OGD/R, and this reduction was restored by dexmedetomidine treatment (Figure 6B). ChIP‑qPCR assays demonstrated the direct binding of TCF4 to the promoter regions of *CLDN5* and *CDH5* in vitro (Figure 6C). A dual‑luciferase reporter assay further verified that TCF4 potently activated the promoters of *CLDN5* and *CDH5* (Figure 6D).

The functional necessity of TCF4 for the barrier‑protective effect of dexmedetomidine was then rigorously examined. In TCF4‑knockout (TCF4‑KO) HUVECs (validation data shown in Figure S6C), the ability of dexmedetomidine to restore VE‑cadherin and Claudin5 protein expression under OGD/R was abolished (Figure 6E). Consequently, dexmedetomidine‑mediated improvement in endothelial barrier function, reflected in reduced permeability to FD70 and increased TER, was lost in TCF4‑KO cells (Figure 6F,G).

In vivo, TCF4 expression was silenced by siRNA technique (si‑TCF4, Figure S6D). Knockdown of TCF4 markedly attenuated the therapeutic efficacy of Dex‑NPs against GVB damage following intestinal I/R. In si‑TCF4 mice, Dex‑NPs failed to upregulate Claudin5 and VE‑cadherin expression in the intestinal vasculature (Figure 6I,J). Consequently, the protective outcomes associated with Dex‑NPs treatment, including reduced PV‑1 levels, decreased hepatic bacterial translocation, and lowered plasma FD70, ALT, and AST levels, were also markedly diminished (Figure S6B and Figure 6J–M). Consistently, Chiu's histological scoring showed that TCF4 knockdown largely abolished the protective effect of Dex‑NPs on intestinal mucosal injury (Figure S6E).

Collectively, these findings demonstrate that dexmedetomidine upregulates TCF4, which directly binds to and activates the promoters of *CLDN5* and *CDH5*, thereby increasing Claudin5 and VE‑cadherin expression and ultimately protecting the GVB after intestinal I/R.

### 2.6 Dexmedetomidine Upregulates TCF4 Expression by Inhibiting HDAC Activity and Promoting H3K27 Acetylation at Its Promoter

**FIGURE 7:**
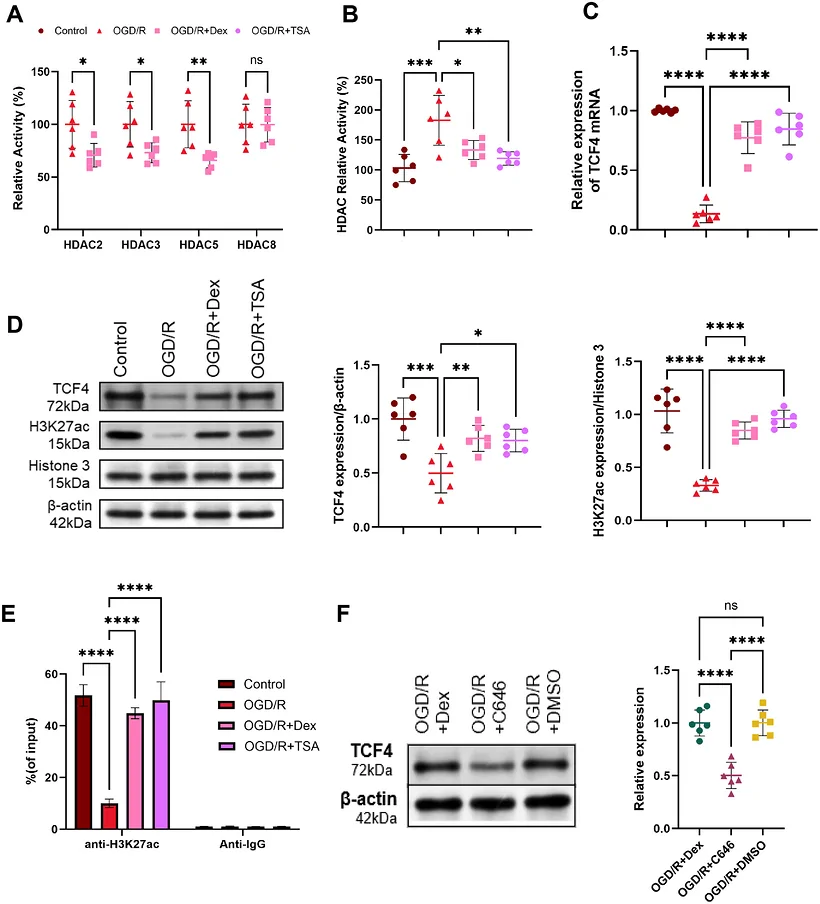
Dexmedetomidine upregulates TCF4 expression by inhibiting HDAC activity and promoting H3K27 acetylation at the TCF4 promoter. (A) Isoform‑specific HDAC activity assays in HUVECs under OGD/R and OGD/R + Dex conditions. (B–E) HUVECs were treated under the following conditions: Control, OGD/R, OGD/R + Dex, and OGD/R + TSA (a known HDAC inhibitor). (B) Relative HDAC activity in each group. (C) qPCR analysis of TCF4 mRNA expression. (D) Western blot analysis of TCF4, H3K27ac, and total histone H3 protein levels. (E) ChIP assay showing enrichment of H3K27ac on the TCF4 promoter. (F) Western blot analysis and quantification of TCF4 protein levels in HUVECs treated with OGD/R + Dex, OGD/R + C646 (a p300/CBP histone acetyltransferase inhibitor), or OGD/R + DMSO. Data are presented as mean ± SD (*n* = 6). ^*^ *p* < 0.05, ^**^ *p* < 0.01, ^***^ *p* < 0.001, ^****^ *p* < 0.0001; ns, not significant. Dex, Dexmedetomidine; TSA, Trichostatin A; HUVECs, Human Umbilical Vein Endothelial Cells; OGD/R, Oxygen‐Glucose Deprivation/Reperfusion; HDAC, Histone Deacetylase; H3K27ac, Acetylated Histone H3 Lysine 27; ChIP, Chromatin Immunoprecipitation; C646, p300/CBP inhibitor; DMSO, Dimethyl Sulfoxide.

Given prior evidence that dexmedetomidine inhibits histone deacetylase (HDAC) activity [27, 28, 29], we investigated its potential role in epigenetic regulation to explore the upstream mechanism by which it enhances TCF4 expression. To identify the specific HDAC isoforms involved in dexmedetomidine‑mediated epigenetic regulation, we performed isoform‑specific activity assays for HDAC2, HDAC3, HDAC5, and HDAC8. The results showed that dexmedetomidine significantly inhibited the activities of HDAC2, HDAC3, and HDAC5, while HDAC8 remained unaffected, suggesting a multi‑isoform synergistic inhibition (Figure 7A). We next examined its effect on total HDAC activity. In HUVECs subjected to OGD/R, HDAC activity was significantly increased relative to the control, and this increase was markedly suppressed by dexmedetomidine to a level comparable to that achieved with the known HDAC inhibitor trichostatin A (TSA) (Figure 7B). Furthermore, similar to dexmedetomidine, TSA increased TCF4 mRNA and protein expression under OGD/R (Figure 7C,D).

Inhibiting HDAC activity is known to increase histone acetylation levels, thereby modulating gene expression. Using the UCSC Genome Browser, we performed bioinformatics analysis to predict the enrichment of common histone acetylation marks at the *TCF7L2* gene (encoding TCF4) promoter. Based on these predictions and related literature, H3K27 acetylation (H3K27ac) was selected for further investigation (Figure S7A–D). We then asked whether dexmedetomidine upregulates TCF4 expression by promoting H3K27 acetylation.

In HUVECs subjected to OGD/R, we found that the stress markedly inhibited intracellular H3K27ac, while dexmedetomidine and TSA induced comparable and significant increases in H3K27 acetylation (Figure 7D). Moreover, ChIP‐qPCR assays revealed significant enrichment of H3K27ac at the *TCF7L2* promoter under basal conditions, which was markedly reduced by OGD/R stress. Both dexmedetomidine and TSA treatments effectively restored H3K27ac enrichment at this locus (Figure 7E), whereas C646, a selective inhibitor of the histone acetyltransferases p300/CBP, abolished the dexmedetomidine‐induced increase in TCF4 expression (Figure 7F).

Collectively, these data demonstrate that dexmedetomidine upregulates TCF4 expression by inhibiting HDAC activity, which in turn promotes H3K27 acetylation at the TCF4 promoter, leading to enhanced transcriptional activation of the gene.

## 3 Discussion

This study establishes a nanoplatform‐based strategy for targeted, low‐dose delivery of dexmedetomidine to the ischemic intestine, protecting the GVB while circumventing systemic toxicity. Mechanistically, we reveal that dexmedetomidine restores Claudin5 and VE‐cadherin expression through an HDAC‐H3K27ac‐TCF4 axis, identifying a previously unrecognized epigenetic transcriptional pathway for GVB protection.

Intestinal I/R injury is a life‐threatening condition that disrupts the intestinal barrier. In this study, we demonstrate for the first time the disruption of the GVB in clinical specimens from patients with intestinal I/R. Furthermore, both in vivo and in vitro experiments showed that this disruption increases vascular permeability, leading to translocation of enteric bacteria and macromolecules into the bloodstream. Based on our previous work [13], we specifically aimed to repair the damaged endothelial junctions of the GVB through dexmedetomidine‑based intervention. However, the dosing regimens employed vary considerably, with murine doses frequently exceeding 25 µg/kg [30] and reaching up to 400 µg/kg [31]. Our previous study reported that in rats, a dose of 10 µg kg^−^^1^ h^−^^1^ of dexmedetomidine induced severe hemodynamic instability [14]. Consistent with previous reports, Yeo et al. found that high‐dose dexmedetomidine (30 µg/kg, i.p.) significantly reduced systolic blood pressure and heart rate in mice, whereas lower doses (3 and 10 µg/kg) did not produce such effects [17]. In critically ill patients, dexmedetomidine reduces the risk of delirium and shortens the duration of mechanical ventilation compared with traditional sedatives, however, its clinical application remains constrained by dose‐dependent bradycardia and hypotension [32]. Consequently, the safety concerns associated with higher doses have consistently impeded the broader clinical use of dexmedetomidine. These findings highlight the urgent need for a strategy that achieves targeted, low‑dose delivery of dexmedetomidine to the injured GVB, preserving therapeutic efficacy while circumventing dose‑limiting toxicity.

Various nanomedicine strategies, including ROS‐scavenging [33, 34], drug‐delivery [35, 36], and protein‐clearing platforms [37, 38], have recently been explored for organ I/R injury. Given that intestinal injury is pathologically featured by overproduction of ROS and upregulation of pro‐inflammatory cytokines, ROS‐responsive nanotherapies have been increasingly employed in the management of various intestinal disorders [39, 40, 41]. We hypothesized that targeted drug delivery nanosystems, among these approaches, could address the dose‐limiting toxicity of high‐dose dexmedetomidine. Therefore, we encapsulated dexmedetomidine within ROS‐responsive polymeric nanoparticles (Dex‐NPs), representing, to our knowledge, the first dexmedetomidine‐loaded nanocarrier designed for GVB repair following intestinal I/R. Using DiR‑labeled nanoparticles, we tracked the in vivo biodistribution of our nanocarriers and directly visualized their preferential accumulation in ROS‑rich ischemic tissues, including the intestine and kidney. Furthermore, we demonstrated that Dex‑NPs released nearly 50% of the encapsulated dexmedetomidine upon ROS stimulation, confirming efficient ischemia‑ specific, ROS‑triggered drug release. The pharmacokinetic advantage of Dex‑NPs was further supported by LC‑MS/MS quantification, which demonstrated that 5 µg/kg Dex‑NPs achieved effective drug enrichment in the intestine while maintaining significantly lower systemic exposure compared with the 20 µg/kg free drug. This tissue‑enrichment effect, combined with the ROS‑responsive release profile, explains how the nanoparticle formulation converts a therapeutically ineffective low dose into an effective one, while circumventing the dose‑limiting cardiorespiratory depression associated with high‑dose free dexmedetomidine. Consequently, Dex‑NPs carrying only 5 µg/kg dexmedetomidine achieved GVB protection comparable to that of high‑dose free drug, without detectable cardiopulmonary side effects. This strategy effectively decouples therapeutic efficacy from systemic toxicity. Notably, blank nanoparticles failed to confer any protection, indicating that ROS scavenging alone is insufficient to restore endothelial junctional integrity and underscoring the essential role of dexmedetomidine‐mediated barrier repair. Given that vascular barrier disruption plays a pivotal role in the pathogenesis of I/R injury across multiple organs [42, 43, 44], Dex‐NPs, by preserving the vascular endothelial barrier, may offer therapeutic benefits extending beyond the intestine to the heart, lung, and kidney. More importantly, polymeric nanocarriers are already under clinical investigation [45], and dexmedetomidine is widely used in clinical practice. Thus, the Dex‑NPs presented herein may represent a promising nanoplatform‑based strategy for protecting vascular integrity against I/R injury, although further development is needed before clinical translation.

Accumulating evidence over recent years has firmly established that dexmedetomidine exerts potent organ protection against I/R injury. This protective effect is largely ascribed to the suppression of pro‑inflammatory cytokine release, the blockade of apoptotic signaling cascades, and the attenuation of excessive oxidative stress [46, 47, 48]. Extending these findings, the mechanistic pathway of dexmedetomidine‑mediated GVB protection elucidated in the present study provides new insights into endothelial barrier regulation. We reveal a previously unrecognized role of TCF4 as a direct transcriptional activator of *CLDN5* and *CDH5* in endothelial barrier regulation. Although TCF4 is known to regulate endothelial cell identity and function [49], its involvement in vascular barrier repair has not been previously reported. Dexmedetomidine upregulated TCF4 expression, and TCF4 silencing via CRISPR/Cas9 knockout or siRNA knockdown markedly attenuated the restoration of Claudin5 and VE‐cadherin, as well as the accompanying improvement in barrier function both in vitro and in vivo. Upstream of TCF4, dexmedetomidine inhibited I/R‐induced HDAC activation, thereby restoring H3K27 acetylation at the *TCF7L2* promoter and driving TCF4 transcription. These findings delineate a novel HDAC‐H3K27ac‐TCF4‐Claudin5/VE‐cadherin axis that not only translates the pharmacological action of dexmedetomidine into endothelial barrier repair but also expands the therapeutic potential for GVB protection. Interventions targeting key nodes along this signal axis, such as selective HDAC inhibitors or TCF4 stabilizers, may recapitulate the barrier‐protective benefits of dexmedetomidine while circumventing α_2_‐adrenergic receptor‐mediated cardiovascular side effects. Specifically, this study establishes TCF4 as an important regulator of vascular endothelial protection following I/R injury. Of note, this axis is proposed as an endothelial‑specific mechanism rather than a universal pathway of dexmedetomidine action. Nevertheless, the delivery of small‑molecule drugs or proteins to tissues with excessive ROS production (i.e., I/R injury, infection and diabetes) via ROS‑responsive nanocarriers to activate TCF4 may offer a potential therapeutic strategy for restoring vascular barrier integrity.

**SCHEME 1:**
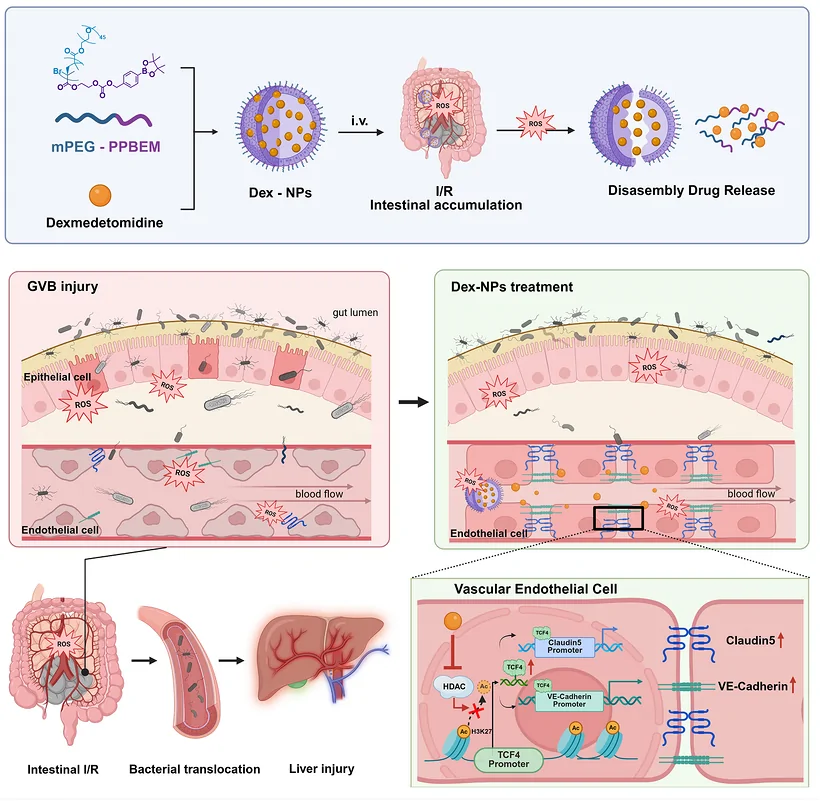
Proposed mechanism of Dex‐NPs in protecting the GVB after intestinal I/R injury. Created by BioRender. Dex‐NPs, dexmedetomidine‐loaded nanoparticles; I/R, intestinal ischemia/reperfusion; GVB, gut vascular barrier.

Several limitations of this study should be acknowledged. First, the control group comprised patients with early‑stage colon cancer rather than healthy individuals, which was necessitated by ethical constraints. Although we sampled macroscopically normal mucosa distant from the tumor, we cannot fully exclude the possibility that this control group may have influenced baseline GVB integrity. Second, Dex‐NPs were administered at the onset of reperfusion in our model. We acknowledge that this does not fully reflect clinical practice, where treatment is often initiated after a delay. Evaluating the therapeutic window using delayed administration regimens would be valuable to better inform clinical translation, and we have noted this as an important direction for future studies. Third, although Dex‐NPs showed no acute toxicity or organ injury after short‐term administration, the chronic toxicity, long‐term degradation profiles, and potential immunogenicity of the mPEG‐PPBEM carrier remain to be systematically evaluated. Final, although our isoform‑specific activity assays revealed that dexmedetomidine significantly inhibits HDAC2, HDAC3, and HDAC5 (likely in a synergistic manner), the specific HDAC isoform directly responsible for H3K27 deacetylation at the *TCF7L2* promoter during I/R remains to be identified. The possibility of off‑target HDAC inhibition contributing to the observed effects cannot be excluded. Elucidating which isoform(s) mediate this specific epigenetic regulation would facilitate the development of isoform‑selective therapeutic strategies with improved safety profiles and represents an important direction for future investigation. In summary, this study establishes a ROS‐responsive nanoplatform‐based strategy that enables low‐dose delivery of dexmedetomidine with preferential accumulation in the ischemic intestine, thereby protecting the GVB without the dose‐limiting cardiorespiratory toxicity of high‐dose free drug (as shown in Scheme 1). Mechanistically, we identify a previously unrecognized HDAC‐H3K27ac‐TCF4‐Claudin5/VE‐cadherin signaling axis through which dexmedetomidine restores endothelial barrier integrity. Beyond providing a promising nanomedicine for intestinal I/R, this work suggests that TCF4 may serve as a potential therapeutic target within this epigenetic‐transcriptional pathway, which could offer new therapeutic avenues for diseases characterized by endothelial barrier dysfunction associated with dysregulated oxidative stress.

## 4 Experimental Section

### 4.1 Collection of Human Intestinal Tissue Specimens

The study enrolled patients undergoing intestinal resection at the First Affiliated Hospital of Sun Yat‐sen University between January 2022 and January 2024. The intestinal ischemia/reperfusion (I/R) group (*n* = 6) included patients aged 18–70 years with intestinal ischemia (e.g., due to intestinal volvulus or incarcerated hernia) refractory to conservative treatment and requiring surgical resection of the ischemic intestine. The control group (*n* = 6) comprised patients undergoing resection for early‑stage colon cancer. To ensure anatomical comparability, the resected intestinal segments in the control group were collected from the same anatomical location (e.g., ileum or colon) as the I/R group whenever possible. Approval for this study was granted by the Ethics Committee of the First Affiliated Hospital of Sun Yat‐sen University (No. [2021]810). All participants voluntarily provided written informed consent. After surgery, intestinal specimens were collected from the ischemic margins (I/R group) or from macroscopically normal segments obtained at least 5 cm away from the tumor (Control group). Each patient's tissues were divided into two portions: one was stored at −80°C for frozen sectioning, and the other was postfixed with 2.5% glutaraldehyde and 1% osmium tetroxide (OsO_4_), then embedded in epoxy resin for electron microscopy.

Demographic and clinical data collection. For each enrolled patient, we collected demographic characteristics (age, sex, BMI), clinical parameters (diagnosis, anatomical sampling site, time from symptom onset), and comorbidities. A complete summary of these variables is presented in Table S1.

### 4.2 Data Acquisition and Blinding

All sample processing and data analyses of this study were conducted by investigators blinded to the experimental group assignments. Image acquisition and quantitative analysis were performed independently by different investigators under blinded conditions. Western blot, qPCR, and immunofluorescence data were normalized to respective internal controls prior to statistical analysis.

### 4.3 Animals and Operative Procedures

The current animal protocol has been reviewed and approved by the Animal Trials of the First Affiliated Hospital of Sun Yat‐sen University ([2023]028). Eight‐week‐old male C57BL/6J mice (22–25 g) were purchased from the Laboratory Animal Center of Sun Yat‐sen University. The mice were allowed free access to water and food.

The mice were anesthetized with pentobarbital (50 mg/kg, intraperitoneally), and an intestinal I/R model was established according to our previous study [13]. In brief, a 2‐cm midline laparotomy was performed, and the superior mesenteric artery (SMA) was identified. The SMA was occluded by a non‐crushing microvascular clip for 45 min. Then the clip was removed, and the abdominal incision was sutured. Based on our previous findings related to gut barrier damage, the mice were sacrificed at 4 h after reperfusion, and biological samples were analyzed [13]. During the experimental period, the mice's temperatures were maintained by the heating pad.

For the renal I/R model, a separate cohort of mice underwent the same anesthetic procedure. Through a 2‐cm midline abdominal incision, both renal pedicles were carefully exposed. Bilateral renal pedicles were simultaneously occluded using non‐crushing microvascular clips for 45 min [50]. The mice were sacrificed at 6 h after reperfusion, and biological samples were analyzed. Body temperature was maintained throughout the procedure using a heating pad.

Mice that died before the scheduled endpoint or showed surgical failure (e.g., incomplete occlusion of the SMA) were excluded from the analysis. No other exclusion criteria were applied.

### 4.4 Cell Culture and Oxygen‐Glucose Deprivation/Reoxygenation Model

Human umbilical vein endothelial cells (HUVECs, Sciencell) were grown in endothelial cell culture medium (ECM, 1001, Sciencell), which was supplemented with 5% fetal bovine serum, 1% streptomycin/penicillin solution, and 1% endothelial cell growth supplement. The cells were maintained in a humidified incubator with 5% CO_2_ at 37°C. For subsequent experiments, HUVECs at passages 3–6 were harvested.

To mimic I/R injury, an oxygen‐glucose deprivation/reoxygenation (OGD/R) model was established. Specifically, HUVECs were incubated in glucose‐free Dulbecco's Modified Eagle Medium (DMEM, 11966025, Gibco) within an incubator gassed with 94% N_2_, 5% CO_2_, and 1% O_2_ at 37°C. After a 2‐h exposure to these conditions, the cells were then transferred to a normoxic environment (5% CO_2_ and 95% air) and cultured for 24 h at 37°C in ECM containing glucose.

### 4.5 Preparation of Dexmedetomidine Loaded Nanoparticles (Dex‐NPs)

The synthesis process of the mPEG‐PPBEM copolymer is elaborated upon in the supplementary materials. Afterwards, mPEG‐PPBEM (20 mg) and dexmedetomidine (1 mg) were dissolved in mixed solvent of 2 mL dimethyl sulfoxide (DMSO) and chloroform (v:v = 1:1). Then, emulsifying the mixed solution into distilled water (20 mL) on an ice bath by ultrasonication at 35% power level for 3 min. Then, removing the chloroform using a rotary evaporator, and dialyzing the mixed solution against distilled water for 48 h to remove DMSO. Finally, the solution was filtered through a 220 nm filter and concentrated into 2 mL using Millipore ultrafiltration tubes to obtain nanoparticles (Dex‐NPs). The molecular weight cut‐off (MWCO) of the dialysis bag and ultrafiltration device is 100 kDa. Similarly, blank nanoparticles (B‐NPs) and 1,1'‐Dioctadecyl‐3,3,3',3'‐tetramethyl indotricarbocyanine iodide (DiR) loaded nanoparticles (DiR‐NPs) were prepared without or with DiR instead of dexmedetomidine. DiR‐NPs were prepared for in vivo imaging studies.

#### 4.5.1 In Vitro Drug Release of Micelles

1 mL of Dex‐NPs was diluted in 2 mL PBS solution under corresponding conditions and added in dialysis bag (MWCO = 100 kDa) to dialyze against 30 mL of various solutions, including pH 7.4 PBS containing 100 µM H_2_O_2_. The release test was conducted at 37°C in an incubator shaker of 100 rpm. At specific time points, 3 mL solution outside was collected to measure the concentration of dexmedetomidine afterwards, then replenished with 3 mL of fresh PBS with the same pH. Dexmedetomidine concentration was analyzed by high‐performance liquid chromatography (HPLC). The drug release studies were measured in triplicate for each condition.

#### 4.5.2 The Serum Stability of Micelles

The 1 mL of Dex‐NPs was diluted 10 mL PBS, and 10 mL PBS containing 10% FBS. Then the particle sizes of the samples were detected at different time points by DLS.

#### 4.5.3 Detection of H_2_O_2_ Consumption of Polymeric Vector

The catalase‐mimetic behavior of mPEG‐PPBEM was evaluated according to the Góth assay [51]. In a typical procedure, 20 mL of hydrogen peroxide solution at varying concentrations was combined with 2 mL of the blank polymeric nanoparticle (B‐NPs) solution, in which the PBEM subunit was maintained at 1.0 mM, and incubated at 37 °C. The resulting reaction mixtures contained final concentrations of 0.05, 0.1, or 1 mM H_2_O_2_, 1 mM PBEM unit, and 450 µM ammonium molybdate. At specified time intervals, 1 mL aliquots were withdrawn, and the reaction was quenched by mixing with 1 mL of ammonium molybdate (1 mM). A control experiment was performed under identical conditions using purified water in place of the polymer solution. The remaining hydrogen peroxide formed a stable yellow complex upon reaction with ammonium molybdate, and its concentration was quantified by measuring the absorbance at 375 nm with reference to a pre‐calibrated standard curve. Hydrogen peroxide consumption was determined as the difference in residual H_2_O_2_ levels between the polymer‐containing samples and the polymer‐free control across the different initial H_2_O_2_ concentrations.

### 4.6 Drug Treatment

All animals were randomly assigned to groups, and drug administration was performed by different researchers under blinded conditions.

For dexmedetomidine dose‑response experiments, mice were intravenously injected with either a low dose (5 µg/kg, Dex5), a high dose (20 µg/kg, Dex20) of dexmedetomidine (S3075, Selleck), or an equal volume of PBS (sham and I/R group) at the beginning of reperfusion.

For nanoparticle experiments, mice were intravenously injected with Dex‐NPs (containing Dex at 5 µg/kg), B‐NPs plus low‐dose dexmedetomidine (B‐NPs+Dex5, Dex at 5 µg/kg), or an equal volume of PBS (I/R+PBS group) via the tail vein at the beginning of reperfusion.

### 4.7 Physiological Monitoring

Heart rate and respiration rate were monitored in conscious mice using a MouseOx Plus pulse oximeter (Starr Life Sciences Corp., USA) at baseline (before reperfusion, designated as “pre”) and at 15 min, 1, 2, and 4 h after reperfusion. Survival status of mice was monitored continuously during the first 4 h after reperfusion.

### 4.8 Establishment of TCF4 Knockout (KO) and Knockdown (KD) Models

The generation of the TCF4‑knockout (TCF4‑KO) HUVEC cell line was performed by Guangzhou Jingrui Biotechnology Co. Ltd. using the CRISPR/Cas9 system. Briefly, two guide RNAs (gRNAs) targeting the TCF4 gene were designed, cloned into the lentiCRISPR v2 vector (Addgene, USA), and used to produce lentivirus in HEK293T cells. HUVECs were then transduced with the lentivirus, and stable knockout pools were selected using puromycin (2 µg/mL). The efficiency of TCF4 knockout was confirmed by Western blotting.

For in vivo TCF4 knockdown, mice were intravenously injected with ESC siRNA targeting TCF4 (Si‐TCF4) or a scrambled control (Si‐NC) (Genma, China) at a dose of 20 µg per mouse daily for three consecutive days, after which the mice were subjected to the intestinal I/R procedure and treated with Dex‐NPs as described above. The efficiency of TCF4 knockdown in mouse intestinal tissues was confirmed by Western blotting.

### 4.9 Histological Assessment of Intestinal Injury

Intestinal tissue sections (4 µm) were stained with hematoxylin and eosin (H&E) for histopathological evaluation. Intestinal mucosal injury was assessed using Chiu et al.’s scoring criteria by two independent pathologists blinded to group allocation. A minimum of five randomly chosen fields from each intestinal mucosa were evaluated and averaged to determine the Chiu's score.

### 4.10 Permeability Assay

To assess GVB permeability, an independent cohort of mice was used to avoid potential interference from fluorescein isothiocyanate‐dextran 70 kDa (FD70) with other experimental variables. Briefly, under anesthesia, a 5 cm segment of the terminal ileum was exteriorized and ligated. FD70 (2 mg in PBS, Sigma‑Aldrich) was injected into the intestinal loop 1 h before euthanasia. Blood samples were then collected, plasma was isolated, and FD70 levels were quantified using a fluorescence microplate reader (BioTek).

### 4.11 Fluorescence In Situ Hybridization (FISH)

To assess bacterial translocation to the liver, fluorescence in situ hybridization was performed using a Cy3‑labeled probe (5′‑GCA TAA GCG TCG CTG CCG‑3′) specific for the conserved region of Escherichia coli 16S rRNA. Liver sections (4 µm) were hybridized with the probe, counterstained with DAPI, and imaged under a confocal microscope (LSM710, Carl Zeiss). The number of bacteria within hepatic sinusoids was quantified in at least five random fields per section (200× magnification) using ImageJ software.

### 4.12 Immunofluorescence

Immunofluorescence staining was performed on intestinal tissue sections (4 µm) after fixation, permeabilization, and blocking. For human tissue sections, the primary antibodies used were anti‑PV‑1 (ab321889, Abcam), anti‑VE‑cadherin (14‑1449‑37, Invitrogen), anti‑Claudin5 (35‑2500, Invitrogen), and anti‑CD31 (691575, Progen,). For mouse tissue sections, the primary antibodies were anti‑PV‑1 (ab27853, Abcam,), anti‑VE‑cadherin (14‑1441‑82, Invitrogen), anti‑Claudin5 (35‑2500, Invitrogen,), and anti‑CD31 (ab7388, Abcam). All sections were incubated with the respective primary antibodies overnight at 4°C, followed by fluorophore‑conjugated secondary antibodies for 1 h at room temperature in the dark. Nuclei were counterstained with DAPI. Images were acquired using a fluorescence microscope (DMi8, Leica, Germany). For each sample, at least five randomly selected fields were captured with identical acquisition settings across all groups. Fluorescence intensity was quantified by ImageJ, normalized to DAPI, and averaged per sample. Multiple fields from the same sample were not treated as independent biological replicates.

### 4.13 ROS Detection

ROS levels in tissues were detected using dihydroethidium (DHE) staining (Invitrogen). Frozen tissue sections (10 µm) were incubated with DHE (5 µmol/L in PBS) at 37°C for 30 min in a dark, humidified container. After washing with PBS, sections were counterstained with DAPI. Fluorescence images were captured using a fluorescence microscope (DMi8, Leica, Germany) with appropriate excitation/emission filters (excitation 518 nm, emission 605 nm), and fluorescence intensity was quantified using ImageJ software. For quantitative assessment, ROS levels in intestine and liver homogenates were measured using a Mouse ROS ELISA Kit (Shanghai Enzyme‐linked Biotechnology, China) according to the manufacturer's instructions.

### 4.14 Endothelial Monolayer Permeability Assay In Vitro

To assess endothelial barrier integrity, HUVECs were seeded (1 × 10^5^ cells/well) in 24‑well hanging inserts and allowed to form a confluent monolayer. After receiving the respective treatments for each experimental group, the medium in the upper chamber was replaced with 200 µL of ECM containing 2% FBS and FD70 (1 mg/mL). Following incubation in the dark for 1 h, 100 µL of medium from the lower chamber was collected, and FD70 leakage was quantified by measuring fluorescence intensity (ex/em: 485/535 nm) using a Varioskan LUX multimode microplate reader (Thermo Fisher Scientific, USA).

### 4.15 Transendothelial Electrical Resistance (TER) Measurement

To assess endothelial barrier integrity, HUVECs were seeded at a density of 1  ×  10^5^ cells per well in 24‑well hanging culture plates equipped with permeable inserts and allowed to form a confluent monolayer. After the endothelial barrier was established and the respective experimental treatments were applied, TER values were measured using the Millicell ESR‑2 Cell Electrical Resistance System (Millipore, USA) according to the manufacturer's instructions. Values were normalized to the resistance of blank inserts and expressed as Ω·cm^2^.

### 4.16 Western Blotting

Protein was extracted from intestinal tissues (mouse) and HUVECs using RIPA lysis buffer. The protein concentration was determined using a bicinchoninic acid (BCA) assay. Equal amounts of protein were separated by SDS‑PAGE and transferred onto PVDF membranes. After blocking with 5% nonfat milk, the membranes were incubated overnight at 4°C with primary antibodies. For mouse tissues, the following primary antibodies were used: anti‑TCF4 (#2569, CST, rabbit) and anti‑β‑actin (66009‑1‑Ig, Proteintech, mouse). For HUVECs, the following primary antibodies were used: anti‑VE‑cadherin (ab33168, Abcam, rabbit), anti‑Claudin5 (35‑2500, Invitrogen, mouse), anti‑TCF4 (#2569, CST, rabbit), anti‑acetyl‑H3K27 (ab4729, Abcam, rabbit), anti‑total H3 (ab1791, Abcam, rabbit), and anti‑β‑actin (66009‑1‑Ig, Proteintech, mouse). After washing, the membranes were incubated with HRP‑conjugated secondary antibodies for 1 h at room temperature. The secondary antibodies used were: HRP‑labeled goat anti‑rabbit IgG (SA00001‑2, Proteintech) and HRP‑labeled goat anti‑mouse IgG (SA00001‑1, Proteintech). Protein bands were visualized using a chemiluminescence imager and quantified with ImageJ software. Band intensities were normalized to β‑actin (for VE‑cadherin, Claudin5, and TCF4) or total H3 (for acetyl‑H3K27).

### 4.17 Quantitative Real‐Time PCR (qPCR)

Total RNA was extracted from cells using the EZ‐press RNA Purification Kit (B0004D, EZBioscience). The extracted RNA was then reverse transcribed into cDNA using the Color Reverse Transcription Kit (A0010CGQ, EZBioscience). Quantitative real‐time PCR was performed using the 2× Color SYBR Green qPCR Mix (A0012, EZBioscience) on a Roche LightCycler 480 System. The thermal cycling conditions were as follows: initial denaturation at 95°C for 30 s, followed by 40 cycles of 95°C for 5 s and 60°C for 30 s. Each reaction was run in triplicate. Relative gene expression was calculated using the 2^−ΔΔCt^ method and normalized to the expression of β‑actin. Primer sequences are listed in Table S2.

### 4.18 Histological Analysis and Serum Biochemistry

For histopathological assessment, tissue samples from the heart, liver, spleen, lungs, and kidneys were collected, fixed in 4% paraformaldehyde, embedded in paraffin, sectioned (4 µm), and stained with hematoxylin and eosin (H&E).

For serum biochemistry, blood was collected by cardiac puncture, and serum was separated by centrifugation. The levels of ALT, AST, and BUN were measured with an automatic biochemistry analyzer (Chemray 800, Shenzhen, China).

### 4.19 In Vivo Fluorescence Imaging

To evaluate the targeting ability of nanoparticles, DiR‐labeled nanoparticles (DiR‐NPs) were intravenously injected into sham and intestinal I/R mice. At 1, 2, 4 and 6 h postinjection, whole‐body fluorescence images were acquired using an in vivo imaging system (In‑Vivo Xtreme II, Bruker, Germany). After the final imaging session, mice were sacrificed, and major organs (including liver, spleen, and kidneys) were collected for ex vivo imaging.

### 4.20 Pharmacokinetic Studies

To evaluate the pharmacokinetic profile of Dex‑NPs, intestinal I/R mice received a single intravenous injection of free dexmedetomidine (5 or 20 µg/kg), free dexmedetomidine (5 µg/kg) combined with blank nanoparticles (B‑NPs + Dex5), or Dex‑NPs (5 µg/kg). Blood, intestinal tissue, and liver samples were collected at 1 h postinjection. Dexmedetomidine concentrations in plasma and tissue homogenates were measured using a liquid chromatography‑tandem mass spectrometry (LC‑MS/MS) system (AB Sciex 4000, USA), with an internal standard method for quantification.

### 4.21 Cell Viability Assay

HUVECs were seeded in 96‐well plates and subjected to OGD/R with various concentrations of Dex‐NPs. Cell viability was assessed using the CCK‐8 assay (C0037, Beyotime) according to the manufacturer's instructions. The absorbance at 450 nm was measured using a microplate reader (Thermo Fisher Scientific, USA).

### 4.22 HDAC Activity Assay

Total HDAC activity and isoform‑specific activities of HDAC2, HDAC3, HDAC5, and HDAC8 in HUVECs were measured using fluorometric assay kits (Abcam for total HDAC; BPS Bioscience for isoform‑specific HDACs), following the manufacturers’ protocols. Fluorescence intensity was read on a microplate reader (Thermo Fisher Scientific, USA).

### 4.23 Chromatin Immunoprecipitation (ChIP) Assay

ChIP assays were performed using a ChIP Kit (17‐295, Millipore) according to the manufacturer's instructions. Briefly, HUVECs were cross‐linked with 1% formaldehyde, and chromatin was sheared by sonication. Immunoprecipitation was performed using antibodies against TCF4 (C48H11, #2569) or H3K27ac (ab4729). Normal rabbit IgG was used as a negative control. Precipitated DNA was analyzed by qPCR using primers specific for the promoter regions of *CLDN5* (Claudin5), *CDH5* (VE‐cadherin), and *TCF7L2* (TCF4). Primer sequences are provided in Table S3.

### 4.24 Dual‐Luciferase Reporter Assay

The promoter regions of human *CLDN5* and *CDH5* were cloned into the pGL3‐Basic luciferase reporter vector (Axl‐Bio). HUVECs were co‐transfected with the reporter vector, a TCF4‐overexpression plasmid, and the Renilla luciferase control vector (pRL‐TK, Promega) using Hieff Trans Liposomal Transfection Reagent (Yeasen Biotechnology, China). After 48 h, firefly and Renilla luciferase activities were measured using the Dual‐Glo Luciferase Assay System (Promega).

### 4.25 Bioinformatic Prediction of Common Transcriptional Regulators

To identify potential common transcription factors regulating *CLDN5* and *CDH5*, we performed a multi‑platform prediction. First, we queried the GTRD and TFDB databases for experimentally supported regulators. Second, using JASPAR PWMs, we conducted enrichment analysis with PWMEnrich and individual binding site scanning with FIMO (score threshold 80%, *p*‑value < 10^−^^4^) on the promoter regions (−2000 bp to +200 bp relative to the transcription start site) of both genes. Common regulators were defined as the overlap of predictions from all four sources for each gene, followed by integration of the two gene‑specific sets to obtain the final common regulators.

### 4.26 Statistical Analysis

All data are presented as mean ± standard deviation (SD). Statistical analyses were performed using GraphPad Prism 8.0 software (GraphPad, USA). Heart rate and respiratory data were analyzed using two‑way analysis of variance (ANOVA) with repeated measures, followed by Bonferroni posttest. For other data, comparisons between two groups were made using the unpaired Student's t‑test. Comparisons among multiple groups were performed using one‐way ANOVA followed by Tukey's post hoc test. A *p*‐value of less than 0.05 was considered statistically significant. Sample size was estimated based on our previous study [13] and preliminary data showing a 40% reduction in PV‑1 expression between groups, using a two‑sided t‑test with 90% power and α = 0.05, yielding 6 mice per group for the main experimental cohorts, with the exact n values specified in each figure legend.

## Author Contributions

**Hu‐Fei Zhang**: Writing – original draft, methodology, data curation, investigation, conceptualization, validation, formal analysis, visualization. **Jian‐Tong Shen**: conceptualization, methodology, investigation, data curation. **Hua‐Hua Zhang**: investigation, data curation, methodology. **Si‐Qi Gao**: methodology, investigation, data curation. **Ji‐Ao Wang**: visualization, investigation, data curation. **Yue‐Ling Wang**: data curation, visualization, investigation. **Mei‐Ling Li**: investigation. **Zi‐Meng Liu**: conceptualization, writing – review and editing, methodology. **Yi‐Nan Zhang**: conceptualization, methodology, visualization, writing – original draft. **Yan Li**: conceptualization, methodology, investigation, writing – review and editing, funding acquisition. **Xu‐Yu Zhang**: conceptualization, investigation, methodology, writing – review and editing, funding acquisition, writing – original draft.

## Funding

This work was supported by the National Natural Science Foundation of China (No. 82072204), the Natural Science Foundation of Guangdong Province, China (No. 2025A1515012493), and the Open Project Program of State Key Laboratory of Frigid Zone Cardiovascular Diseases (SKLFZCD), Harbin Medical University (HDHY2025032). No artificial intelligence (AI) tools were used in the generation of any data, figures, or images presented in this manuscript. The authors take full responsibility for the integrity and accuracy of all content.

## Conflicts of Interest

The authors declare no conflicts of interest.

## Supporting information




**Supporting File**: advs77702‐sup‐0001‐SuppMat.docx.

## Data Availability

The data that support the findings of this study are available in the supplementary material of this article.
