# Supplementary material for: ROS‐Responsive Nanoparticle Delivery of Dexmedetomidine Protects the Gut Vascular Barrier After Intestinal Ischemia/Reperfusion via the HDAC‐H3K27ac‐TCF4 Axis
Source: Adv Sci (Weinh). 2026 Sep 8:e77702. Online ahead of print. doi: 10.1002/advs.77702 (PMC13554394; doi:10.1002/advs.77702)
Supplement: Supplementary file 1 [file ADVS-9999-e77702-s001.docx]

**Supplementary materials**

**Materials and methods**

**Synthesis of the mPEG-PPBEM Copolymer**

**Materials**

4-Hydroxymethyl benzhydrol, furfural, N,N-carbonyldiimidazole (CDI), dichloromethane, anhydrous MgSO_4_, 4-dimethylaminopyridine (DMAP), hydroxyethyl methacrylate, petroleum ether, ethyl acetate, monomethyl ether polyethylene glycol (mPEG-OH, Mw = 2 kDa), triet hylamine, 2-bromoisobutyryl bromide, anhydrous ether, N,N-dimethylformamide (DMF), N，N，N′，N′，N″-pentamethyldiethylenetriamine (PMDETA), copper(I) bromide, cuprous oxide, methanol, dimethyl sulfoxide (DMSO), dexmedetomidine, deuterated chloroform (CDCl_3_), 10% hydrogen peroxide (H_2_O_2_), fetal bovine serum (10% FBS), phosphate buffered saline (PBS).

**Characterization**

^1^H NMR spectra were obtained at 25 °C on a Bruker 300 MHz spectrometer, using DMSO-*d*_6_ or CDCl_3_ as solvent. FT-IR spectra were recorded on a FTIR spectrometer (Thermo Fisher Scientific) by using KBr slices. The particle size and zeta potential were detected by dynamic light scattering (DLS) measurements at 25 °C (Brookhaven Omni). The data of particle size and zeta potential were collected on an auto-correlator with a detection angle of 173° for scattered light. Transmission electron microscopy (TEM) images were obtained on a JEM 1400Flash (JEOL). The samples were stained with uranyl acetate aqueous solution (1.0 wt %) for 1 min and dried on a copper grid.

**The critical micelle concentration (CMC) detection**

A solution of pyrene in benzene at a concentration of 2 × 10⁻⁵ mol/L and was prepared. The polymer mPEG-PPBEM was dissolved in deionized water at a concentration of 2 mg/mL. Aliquots of the pyrene solution (50 μL) were added into 10 mL volumetric flasks, and the benzene was allowed to evaporate naturally. Polymer solutions and PBS (pH 7.4) were then added to obtain a series of polymer concentrations of 0.1, 0.5, 1, 2, 4, 5, 10, 20, 100, and 1000 μg/mL. The flasks were placed in an ultrasonic bath and shaken for 30 min. The fluorescence spectra of each solution were recorded. Fluorescence scanning was performed at an excitation wavelength of 334 nm, with excitation and emission slit widths both set at 3.0 nm. The intensity ratio I₃₇₃/I₃₈₄ (I₁/I₃) was plotted against the polymer concentration to obtain the CMC.

**Synthesis of CDI activated pinacol boronic ester**

4-(Hydroxymethyl) phenylboronic acid (3.0 g, 19.7 mmol), pinacol (2.8 g, 23.8 mmol) and sodium sulfate (3.0 g) were dissolved in THF (30 mL) solvent. After the reaction was kept stirring at room temperature for 12 h under nitrogen protection, the mixture was filtered, concentrated, re-dissolved in ethyl acetate and precipitated into excessive cold diethyl ether. The precipitate was filtered, washed with diethyl ether twice, and vacuum-dried to obtain the product 4-(hydroxymethyl)phenylboronic acid pinacol ester (yield: 72.3%). Subsequently, under the protection of nitrogen, 4-(hydroxymethyl)phenylboronic acid pinacol ester (2.5 g, 10.6 mmol), and CDI (3.0 g, 48.6 mmol) were added into a 100 mL Schlenk bottle and dissolved using 10 mL dichloromethane (CHCl_3_). After the solution was stirred at 25 °C for 24 h, adding 20 mL of dichloromethane (CHCl_3_) to dilute the solution. Then, the obtained mixture was washed with pure water three times, collecting the organic layer liquid and adding anhydrous MgSO_4_ to remove residual water. After filtration, the solvent was evaporated by rotary evaporation, a white powder product CDI activated pinacol boronic ester was obtained (2.7 g, 80%).

**Synthesis of monomer 2-((((4-(4,4,5,5-tetramethyl-1,3,2- dioxaborolan-2-yl)benzyl)oxy)carbonyl)oxy)ethyl methacrylate (PBEM)**

Under nitrogen protection, the CDI-pinacol boronic ester (2 g, 6.1 mmol), 4-dimethylaminopyridine (DMAP, 10 mg, 0.08 mmol) and hydroxyethyl methacrylate (1.3 g, 10.3 mmol) were mixed in 100 mL Schlenk flasks and dissolved in 20 mL CHCl_3_, and stirred for 12 h at room temperature. After reaction, the mixture was concentrated by rotary steaming, and the concentrated solution was then purified by chromatography column. The eluent was mixed solution of ethyl acetate and petroleum ether (V: V = 1:10). Finally, after collecting and purifying the collected solution by rotary evaporation, a yellow powder of PBEM monomer was obtained (1.4 g, 58%).

**Synthesis of chain transfer agent PEG-Br**

2.0 g (1.0 mmol) of monomethyl ether polyethylene glycol (*m*PEG-OH, *M*w = 2 kDa) was dissolved in 10 mL dichloromethane (CHCl_3_). The solution was placed on an ice bath, 417 μL (3 mmol) of triethylamine was added and kept stirring for 15 min. After 378 μL (3 mmol) of 2-bromoisobutyrylbromide was quickly added, the reaction was sealed and stirred for 2 h on ice bath and allowed for another 36 h at room temperature. After filtering the solution, triethylamine salt was removed. The filtrate was concentrated by rotary evaporation, and the obtained solution was precipitated into ice anhydrous ether. After the mixture was left to stand overnight, it was filtered to obtain the filter cake, which was dried under vacuum at 37 °C to obtain white solid *m*PEG-Br.

**Synthesis of *m*PEG-PPBEM**

*m*PEG-PPBEM was synthesized by ATRP polymerization using PEG-Br as macromolecular initiator, with PMDETA/CuBr as catalytic system, and DMF as solvent. Briefly, *m*PEG-Br (0.215 g, 0.1 mmol), PBEM (1.2 g, 0.087 mmol), PMDETA/CuBr (3 eq.) were added to Schlenk flask dissolved in 30 mL DMF. After three cycles’ freeze thawing to remove oxygen, cuprous oxide was added and the solution was stirred for 48 h at 75 °C. After reaction, cuprous oxide was added and stirred for 30 min under open conditions. Then excessive PMDETA was added and dialyzed against methanol for 2 days and deionized water for 5 days to remove Cu ions. The solution was then freeze-dried to obtain white solid *m*PEG-PPBEM (1.1 g, 78%).

***In vitro* drug release of micelles**

1 mL of Dex-NPs was diluted in 2 mL PBS solution under corresponding conditions and added in dialysis bag (MWCO = 100 kDa) to dialyze against 30 mL various solutions, including pH 7.4 PBS containing 100 μM H2O2. The release test was conducted at 37 °C in an incubator shaker of 100 rpm. At specific time points, 3 mL solution outside was collected to measure the concentration of Dex afterwards, then replenished with 3 mL fresh PBS with the same pH. Dex concentration was analyzed by high performance liquid chromatography (HPLC). The drug release studies were measured in triplicate for each condition.

**The serum stability of micelles**

1 mL of Dex-NPs was diluted 10 mL PBS, and 10 mL PBS containing 10% FBS. Then the particle sizes of samples were detected at different time points by DLS.

**Detection of H_2_O_2_ consumption of polymeric vector**

The catalase-mimetic behavior of mPEG-PPBEM was evaluated according to the Góth assay. In a typical procedure, 20 mL of hydrogen peroxide solution at varying concentrations was combined with 2 mL of the blank polymeric nanoparticle (B-NPs) solution, in which the PBEM subunit was maintained at 1.0 mM, and incubated at 37 °C. The resulting reaction mixtures contained final concentrations of 0.05, 0.1, or 1 mM H₂O₂, 1 mM PBEM unit, and 450 µM ammonium molybdate. At specified time intervals, 1 mL aliquots were withdrawn and the reaction was quenched by mixing with 1 mL of ammonium molybdate (1 mM). A control experiment was performed under identical conditions using purified water in place of the polymer solution. The remaining hydrogen peroxide formed a stable yellow complex upon reaction with ammonium molybdate, and its concentration was quantified by measuring the absorbance at 375 nm with reference to a pre-calibrated standard curve. Hydrogen peroxide consumption was determined as the difference in residual H₂O₂ levels between the polymer-containing samples and the polymer-free control across the different initial H₂O₂ concentrations.

***In vivo* clearance of polymeric carrier**

The salicyl fluoresone containing o-phenolic hydroxyl groups was used to label mPEG-PPBEM, and the labeled polymer self-assembled into SF-NPs. The SF-NPs were injected into the tail vein of C57BL/6J mice (n = 3). The orbital blood of 400 μL were obtained at different time points (0 h, 0.5 h, 1 h, 2 h, 4 h, 6 h, 12 h and 24 h) after injection, and were placed in the EDTA blood anticoagulant tubes to prevent solidification. The fluorescence intensity of salicyl fluoresone in the blood was detected by fluorescence spectrophotometer after a 10-fold dilution. A control mouse were analyzed as well for data calibration. The declined percentages of salicyl fluoresone fluorescence intensities represent the rates of polymer clearance from blood.

**Figure S1.** **Effects of different doses of dexmedetomidine on gut vascular barrier (GVB), intestinal mucosal injury, and acute survival.**

Mice were treated with PBS (Sham/Ischemia/Reperfusion (I/R)) or different doses of dexmedetomidine (Dex 5, 5 µg/kg; Dex 20, 20 µg/kg) and assessed 4 h after reperfusion. (A) Representative immunofluorescence images of ileum sections showing the endothelial marker CD31 (red), the vascular permeability marker PV-1 (green), and nuclei (DAPI, blue). Scale bar: 100 μm. (B) Survival of mice within 4 hours after reperfusion. (C) Representative H&E-stained sections of ileum and quantitative Chiu’s histological scoring of intestinal mucosal injury in each group. Scale bar: 100 μm. Data are presented as mean ± SD (n = 6). ****p* < 0.001, *****p* < 0.0001; ns, not significant.

**Figure S2. Intestinal I/R injury specifically induces a marked increase in intestinal reactive oxygen species (ROS) generation.**

(A) Immunofluorescence staining showing ROS production (red) in the kidney, lung, spleen, liver, heart, and intestine of sham and I/R injured mice. DAPI (blue). Scale bar: 100 μm. (B) Quantitative analysis of ROS fluorescence intensity from the immunofluorescence images shown in (A). (C) ELISA-based quantitative analysis of ROS levels in intestinal and hepatic tissue homogenates. Data are presented as mean ± SD (n = 6). *****p* < 0.0001; ns, not significant.

**Figure S3. Synthesis and characterization of mPEG-PPBEM and ROS-responsive nanoparticles.**

(A) Synthetic approach for the synthesis of mPEG-PPBME. ^1^H Nuclear Magnetic Resonance (NMR) spectrum of (B) CDI-PBE in DMSO-d6, (C) PBEM monomer in CDCl_3_, (D) PEG-Br in CDCl3, and (E) mPEG-PPBEM in CDCl_3_. (F) Fourier Transform Infrared (FT-IR) spectra of mPEG-PPBEM. The characteristic vibrational absorption peaks of PPBEM at 1740 cm−1 (ν C=O, ester), 745 cm−1 and 696 cm−1 (δ Ph-H). (G) Zeta (ζ) potential of B-NPs, Dex-NPs, and Dex-NPs plus 0.1 mM ROS (n = 3). “ND” means “Not detected”. (H) Fluorescence intensity ratio (I₃₇₃/I₃₈₄) of pyrene in various mPEG‑PPBEM concentrations. The CMC was determined to be 4.3 μg/mL in aqueous solution. (I) Blood clearance of polymer measured from the salicyl fluoresone fluorescence intensity of the orbital blood after tail vein injection of SF‑NPs. Dose: 2.0 mg polymer/kg body weight. Data are presented as mean ± SD (n = 3). Time point of 0 h was normalized as 100%.

**Figure S4. *Ex vivo* fluorescence imaging of organs and LC‑MS/MS chromatograms.**

(A) *Ex vivo* fluorescence imaging of kidneys and spleens harvested from sham‑operated and intestinal I/R mice at 6 h post‑reperfusion, with quantitative analysis of fluorescence signals. (B) *Ex vivo* fluorescence imaging of kidneys from a separate renal I/R model (sham‑operated vs. I/R) at 6 h post‑reperfusion, with quantitative analysis of fluorescence signals. Data are presented as mean ± SD (n = 3). ***p* < 0.01; ns, not significant. (C) Representative LC‑MS/MS chromatograms: (C1) internal standard-qualitative ion; (C2) internal standard-quantitative ion; (C3) dexmedetomidine-qualitative ion; (C4) dexmedetomidine-quantitative ion.

**Figure S5. Effects of different nanoparticle formulations on gut vascular barrier,** **intestinal mucosal injury, acute survival, and physiological parameters.**

Mice subjected to intestinal I/R were treated with PBS (I/R+PBS), Dex-NPs, or B-NPs + Dex 5. (A) Representative immunofluorescence images of ileum sections stained for CD31 (red), PV-1 (green), and nuclei (DAPI, blue). Scale bar: 100 μm. (B) Survival of mice within 4 hours after reperfusion. All groups maintained 100% survival. (C-D) Time courses of (C) heart rate and (D) breath rate monitored during the reperfusion period. No significant differences were observed among the three groups (n = 6). (E) Representative H&E‑stained sections of ileum and quantitative Chiu’s histological scoring of intestinal mucosal injury in each group. Scale bar: 100 μm. Data are presented as mean ± SD (n = 6). ***p* < 0.01; ns, not significant.

**Figure S6. Identification of TCF4 as a key transcription factor and verification of TCF4 knockdown/knockout.**

(A) Bioinformatics screening for transcription factors (TFs) binding to the promoters of *CDH5* and *CLDN5*. Venn diagrams show the intersection of predicted TFs from four databases (GTRD, PWMEnrich_JASPAR, FIMO_JASPAR, TFDB). Five common TFs (ZNF770, EBF1, EBF3, ESR2, and TCF4) were identified in the overlapping region.

(B) Representative immunofluorescence images of ileum sections stained for CD31 (red), PV-1 (green), and nuclei (DAPI, blue). Mice were treated with si-NC or si-TCF4 followed by I/R injury. Scale bar: 100 μm.(C) Western blot analysis of TCF4 protein expression in wild type (WT) and TCF4-Knockout (TCF4-KO) mice. (n = 3). (D) Western blot analysis of TCF4 protein expression in intestinal tissues from mice treated with si-NC or si-TCF4 (n = 3). (E) Representative H&E‑stained sections of ileum and quantitative Chiu’s histological scoring of intestinal mucosal injury in si‑NC and si‑TCF4 mice. Scale bar: 100μm. Data are presented as mean ± SD (n = 6). ****p* < 0.001; ns, not significant.

**Figure S7. H3K27ac enrichment at the TCF4 genomic locus.**

(A) H3K27ac ChIP‑seq data from human umbilical vein endothelial cells identified TCF4 as a potential target gene (score 5.019). (B) UCSC Genome Browser view showing H3K27ac ChIP‑seq signals (black peaks) co‑localizing with the TCF4 gene locus (red boxes). (C) Prediction of H3K27 acetylation sites in the TCF4 promoter region. H3K27ac enrichment peaks (blue histogram) are located within the regions indicated by red boxes. (D) ChIP‑qPCR validation of H3K27ac enrichment at three primer regions within the TCF4 locus. The anti‑H3K27ac group showed significantly higher % of input compared to the IgG control group (n = 3, ***p* < 0.01, ****p* < 0.001). The primer sequences are as follows:

Primer 1:

Sense: AGAAGCGGCAACACGAATAA

Anti‑sense: GGCGACCATAGAGTGGTAAAC

Primer 2:

Sense: CGTTTCAATTTGCCGTCAGAG

Anti‑sense: AGCGTGCTTTATCCGGTATATG

Primer 3:

Sense: ATTGTAACTGACGATCTGAGGAAA

Anti‑sense: CCCTGACTCTTAACACCAACTC

**Table S1 Baseline demographic, clinical, and laboratory characteristics of control and I/R patients**

| **Variables**  **Groups** | **Age**  **(years)** | **Sex** | **BMI (kg/m^2^)** | **Diagnosis** | **Time from symptom onset** | **Comorbidities** | **Sampling site** |
| --- | --- | --- | --- | --- | --- | --- | --- |
| Control (Human, n=6) | | | | | | | |
|  | 57 | Female | 23 | Ascending colon cancer | - | Hypertension | Ileum |
|  | 43 | Male | 23.8 | Ascending colon cancer | - | - | Ileum |
|  | 70 | Male | 20 | Ascending colon cancer | - | - | Ileum |
|  | 66 | Male | 22 | Ascending colon cancer | - | Hypertension, Diabetes | Ileum |
|  | 44 | Female | 24.45 | Ascending colon cancer | - | Post‑cesarean section | Ileum |
|  | 56 | Female | 23 | Ascending colon cancer | - | Post‑appendectomy | Ileum |
| I/R (Human, n=6) | | | | | | | |
|  | 60 | Female | 21 | Strangulated intestinal obstruction | 2d | Post‑appendectomy | Ileum |
|  | 63 | Male | 20.2 | Superior mesenteric artery embolism | 1d | Hypertension | Ileum |
|  | 62 | Female | 26 | Strangulated femoral hernia | 2d | Diabetes | Ileum |
|  | 73 | Female | 19.8 | Incarcerated obturator hernia | 15h | Hypertension | Ileum |
|  | 56 | Female | 20 | Volvulus | 2h | Post‑myomectomy | Ileum |
|  | 40 | Male | 27 | Superior mesenteric artery embolism | 2d | - | Ileum |

**Table S2 Primers used for quantitative real-time PCR**

| Primer Name | Sequence (5'-3') |
| --- | --- |
| actin-beta F | CATGTACGTTGCTATCCAGGC |
| actin-beta R | CTCCTTAATGTCACGCACGAT |
| TCF4 F | CAAGCACTGCCGACTACAATA |
| TCF4 R | CCAGGCTGATTCATCCCACTG |
| ZNF770 F | CCTCAATACCGCCAAGGTCT |
| ZNF770 R | GTTGCCTCAAGGCTGAAAGC |
| EBF1 F | GGGGGAGGAGATTTTCCACA |
| EBF1 R | AGACCCACCCCGCTCTG |
| EBF3 F | CCAACGACCAAGTCGGCTAC |
| EBF3 R | CGTAGGGAGAGTTAGCGGAG |
| ESR2 F | CCGATGCTTTGGTTTGGGTG |
| ESR2 R | TCCATGCCCTTGTTACTCGC |

**Table S3 Primers used for ChIP quantitative PCR**

| Primer Name | Sequence (5‘→3’) |
| --- | --- |
| *TCF7L2*-promoter-F | ATTGTAACTGACGATCTGAGGAAA |
| *TCF7L2*-promoter-R | CCCTGACTCTTAACACCAACTC |
| *CLDN5*-promoter-F | ACTGGAAACAAAGGGGCCG |
| *CLDN5*-promoter-R | CGAAAGGTCCACTGGTGTG |
| *CDH5*-promoter-F | ATGAGCTGGTCAGTGGCTG |
| *CDH5*-promoter-R | GGCTGGTCTTGAACTCCTGT |
